# Genomic Footprints of Selective Sweeps from Metabolic Resistance to Pyrethroids in African Malaria Vectors Are Driven by Scale up of Insecticide-Based Vector Control

**DOI:** 10.1371/journal.pgen.1006539

**Published:** 2017-02-02

**Authors:** Kayla G. Barnes, Gareth D. Weedall, Miranda Ndula, Helen Irving, Themba Mzihalowa, Janet Hemingway, Charles S. Wondji

**Affiliations:** 1 Vector Biology Department, Liverpool School of Tropical Medicine, Liverpool, United Kingdom; 2 FAS Center for Systems Biology, Department of Organismic and Evolutionary Biology, Harvard University, Cambridge, Massachusetts, United States of America; 3 Broad Institute, Cambridge, Massachusetts, United States of America; 4 Malaria Alert Centre, College of Medicine, University of Malawi, Blantyre, Malawi; 5 Organisation de Coordination pour la lutte contre les Endémies en Afrique Centrale, Yaoundé, Cameroon; The University of North Carolina at Chapel Hill, UNITED STATES

## Abstract

Insecticide resistance in mosquito populations threatens recent successes in malaria prevention. Elucidating patterns of genetic structure in malaria vectors to predict the speed and direction of the spread of resistance is essential to get ahead of the ‘resistance curve’ and to avert a public health catastrophe. Here, applying a combination of microsatellite analysis, whole genome sequencing and targeted sequencing of a resistance locus, we elucidated the continent-wide population structure of a major African malaria vector, *Anopheles funestus*. We identified a major selective sweep in a genomic region controlling cytochrome P450-based metabolic resistance conferring high resistance to pyrethroids. This selective sweep occurred since 2002, likely as a direct consequence of scaled up vector control as revealed by whole genome and fine-scale sequencing of pre- and post-intervention populations. Fine-scaled analysis of the pyrethroid resistance locus revealed that a resistance-associated allele of the cytochrome P450 monooxygenase *CYP6P9a* has swept through southern Africa to near fixation, in contrast to high polymorphism levels before interventions, conferring high levels of pyrethroid resistance linked to control failure. Population structure analysis revealed a barrier to gene flow between southern Africa and other areas, which may prevent or slow the spread of the southern mechanism of pyrethroid resistance to other regions. By identifying a genetic signature of pyrethroid-based interventions, we have demonstrated the intense selective pressure that control interventions exert on mosquito populations. If this level of selection and spread of resistance continues unabated, our ability to control malaria with current interventions will be compromised.

## Author Summary

Malaria control currently relies heavily on insecticide-based vector control interventions. Unfortunately, resistance to insecticides threatens the continued effectiveness of these measures. Metabolic resistance, caused by increased detoxification of insecticides, presents the greatest threat to vector control, yet it remains unclear how these mechanisms are linked to underlying genetic changes driven by the massive selection pressure from these interventions, such as the widespread use of Long Lasting Insecticide Nets (LLINs) across Africa. Therefore, understanding the direction and speed at which this operationally important form of resistance spreads through mosquito populations is essential if we are to get ahead of the ‘resistance curve’ and avert a public health catastrophe. Here, using microsatellite markers, whole genome sequencing and fine-scale sequencing at a major resistance locus, we elucidated the Africa-wide population structure of *Anopheles funestus*, a major African malaria vector, and detected a strong selective sweep occurring in a genomic region controlling cytochrome P450-based metabolic pyrethroid resistance in this species. Furthermore, we demonstrated that this selective sweep is driven by the scale-up of insecticide-based malaria control in Africa, highlighting the risk that if this level of selection and spread of resistance continues unabated, our ability to control malaria with current interventions will be compromised.

## Introduction

Scaling up of malaria prevention and treatment has averted over 660 million cases of malaria since 2000 [1]. The vast majority of this reduction has come from mosquito control with pyrethroid insecticide-based interventions, primarily the use of long lasting insecticide-treated bednets (LLINs) and, to a lesser extent, indoor residual spraying (IRS). Resistance to insecticides in major malaria vectors such as *Anopheles funestus* threatens the continued success of these interventions. Unless resistance is managed, the massive reduction of malaria transmission from scaling up these interventions could be reversed [2]. A key prerequisite for resistance management is to understand the evolution of insecticide resistance to predict the speed and direction of spread of resistance and provide vital information to implement successful control strategies [3]. There are multiple mechanisms of insecticide resistance, these include changes to insecticide target molecules that render the insecticide unable to bind, behavioural changes leading to the avoidance of insecticide contact, thickening of the insect’s cuticle to prevent the insecticide reaching its target and detoxification of the insecticide before it reaches its target (metabolic resistance). Of these, metabolic resistance has the greatest operational significance [3, 4], yet it remains unclear how mosquito populations exhibiting these mechanisms respond to insecticide-based interventions including LLINs. Selective sweeps associated with target-site resistance have been assessed in mosquito species [5, 6] but no such assessment has been made for metabolic resistance. In the major malaria vector *An*. *funestus*, pyrethroid resistance is mainly conferred by metabolic resistance associated with a major quantitative trait locus (QTL) at which two duplicated cytochrome P450 monooxygenases (*CYP6P9a* and *CYP6P9b*) are the main resistance genes [7] [8]. The predominance of metabolic resistance in *An*. *funestus* makes this species very suitable to assess metabolic resistance-based evolutionary responses of mosquitoes to the massive scale up of pyrethroid-based vector control interventions across Africa.

In the context of increasing reports of insecticide resistance in malaria vectors such as *An*. *funestus* across Africa [9–16], it is important to determine the relative contributions to this of gene flow and of the autochthonous appearance of insecticide resistance. Previous studies suggest significant genetic structure among *An*. *funestus* populations across Africa [17]. Whether such differences in genetic structure explain the contrasting insecticide resistance patterns seen in this species [9–16] and could help predict the speed and direction of spread of resistance remains to be determined.

Here, using microsatellite analysis, whole genome sequencing and fine-scale sequencing at a resistance locus, we elucidated the Africa-wide population structure of *An*. *funestus* and detected a strong selective sweep occurring at a major cytochrome P450-based pyrethroid resistance locus. Moreover, we demonstrated that this selective sweep is driven by the scale-up of insecticide-based malaria control in Africa, highlighting the risk that if this level of selection and spread of resistance continues unabated, our ability to control malaria with current interventions could be compromised.

## Results

### 1-African-wide microsatellite diversity shows evidence of positive selection on *An*. *funestus* chromosome 2R

**Fig 1:**
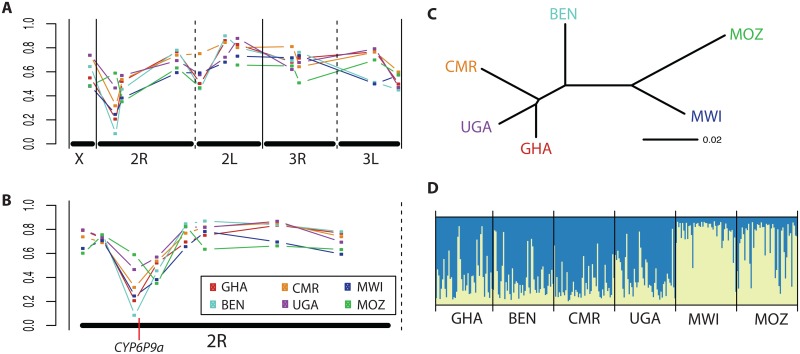
Africa-wide population structure analysis. **A)** Gene diversity of 11 microsatellites from throughout the genome, showing a loss of diversity at the telomeric end of chromosome 2R. **B)** Gene diversity of 8 microsatellites on chromosome 2R (including AFUB6, FunR and FunO) shows that the loss of diversity is restricted to AFUB6 and FunR, the microsatellites located near the pyrethroid resistance QTL *rp1*. **C)** Neighbor-Joining tree based on pairwise *F*_*st*_ among population samples, estimated using 9 neutrally evolving microsatellites from throughout the genome (excluding AFUB6 and FunR on chromosome 2R, which may be evolving under positive selection). **D)** Barplot of assignment probabilities of individual genotypes to two ancestral clusters (the most likely number estimated from the data) estimated using Bayesian population structure analysis under an admixture model. Each bar is an individual genotype for 9 neutrally evolving microsatellites from throughout the genome (excluding AFUB6 and FunR). In panel A, points from left to right represent the following microsatellites: FunQ (on chromosome X); AFUB6, FunR, FunO (on 2R); AFUB11, FunL, AFUB10 (on 2L); AFND7, AFND19 (on 3R); FunF and AFUB12 (on 3L). In panel B, points from left to right represent AFUB3, AFND40, AFUB6, FunR, AFND6, AFND30, AFND32 and FunO (all on 2R).

Analysis of genetic diversity at 11 microsatellites from across the *An*. *funestus* genome sampled in six populations from West (Ghana and Benin), Central (Cameroon), East (Uganda) and southern Africa (Malawi and Mozambique) revealed reduced diversity in two microsatellites at the telomeric end of chromosome arm 2R (Fig 1A). These microsatellites; AFUB6, located in a genomic region spanning a pyrethroid resistance QTL (*rp1)* and FunR, located in the same QTL in the 5' un-translated region of the pyrethroid resistance gene *CYP6P9a* (Fig 1B), showed few alleles and low heterozygosity (S1 Table).

We hypothesised that this low diversity was due to a selective sweep in this QTL region driven by pyrethroid-based vector control interventions. To further localise the putative sweep, we genotyped 5 additional microsatellites upstream and downstream of FunR and AFUB6 on chromosome 2R. This revealed that the reduced diversity was restricted to the two markers within *rp1* (S2 Table). Consistent with our hypothesis, the lowest gene diversity at FunR was seen in the two southern African populations from Malawi and Mozambique (Fig 1A), which also show the highest levels of pyrethroid resistance [4, 18].

#### Continent-wide population structure of *Anopheles funestus*

To investigate the population structure of *An*. *funestus* throughout Africa, we removed the two microsatellites putatively under selection to analyse the remaining 9 neutral markers. Genetic divergence (*F*_*st*_) among countries ranged from 2.9% for Ghana-Uganda to 10.8% for Uganda-Mozambique (S2 Table). Relative divergence among samples from different countries was in broad agreement with their geographical locations, with a major exception: Uganda clustered with Ghana and Cameroon (Fig 1C; *F*_*st*_ 2.9–4.1%) while Benin formed an outlier from the other samples (Fig 1C; *F*_*st*_ 4.9–9.6%). The southern African populations from Malawi and Mozambique were both highly divergent from the other samples (Fig 1C; *F*_*st*_ 6.5–10.8%) though, interestingly, also from each other (6.3%).

The impact of the loci putatively under selection on chromosome 2R was investigated by repeating the analyses on two datasets, one comprising 8 microsatellites on chromosome 2R and one comprising 8 microsatellites on the other chromosomes (S1 Fig). The most striking effect of analysing 2R alone is on Ghana, which becomes highly divergent from all other populations including Benin, the other West African population (S1A Fig; F*st* 14.4–21.9%). When the 8 microsatellites on the other chromosomes are analysed, the pattern of differentiation is more aligned with geographical distance between all samples, with Ghana samples less differentiated from Benin (*F*_*st*_ = 4.3%) than with the 2R markers (*F*_*st*_ = 21.9%) (Fig 1C; S1B Fig). The major divergence between southern Africa and other populations is maintained (S1 Fig). This apparent discontinuity between southern and the rest of Africa is consistent with the contrasting resistance patterns and mechanisms seen among *An*. *funestus* populations [17] suggesting possible barriers to gene flow between regions. This is supported for example by the complete absence throughout southern Africa of the DDT 119F resistance allele of the glutathione-S transferase gene GSTe2, which is predominant in West/Central Africa [15, 19].

Bayesian analysis of population structure was also undertaken on the 9 putatively neutral loci. The most likely number of clusters estimated from the data was 2, and represents the major divergence between southern Africa and elsewhere (Fig 1D). When analyses were repeated using 8 2R microsatellites and 8 non-2R microsatellites, the most likely number of clusters were 3 and 2, respectively. Analysis of the cluster assignment probabilities (to 3 clusters) for all three datasets showed that Malawi and Mozambique were consistently assigned to their own ‘southern’ cluster, even with the 2R marker set (S3 Table). By contrast, the other populations were more variable, and affected by markers on 2R. For instance, using non-2R markers Ghana and Benin were assigned to the same cluster (44% and 41%, respectively), but using 2R markers they were clearly assigned to separate clusters (Ghana cluster 1: 76% and Benin cluster 2: 65%).

Overall, the analyses are consistent in indicating that gene flow is restricted between southern and the rest of Africa. They also suggest that selection on chromosome 2R may be independent among different geographical regions but appears to be common to both southern African *An*. *funestus* populations.

### 2-Fine scale genomic sequence analysis confirms a selective sweep associated with pyrethroid resistance

#### Patterns of polymorphism across the 120kb *rp1* genomic region

**Fig 2:**
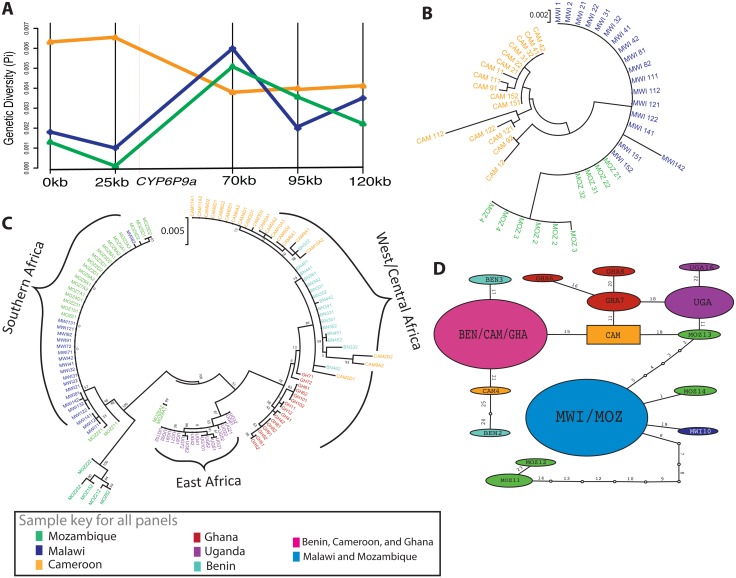
Africa-wide analysis of genetic diversity across the *rp1* pyrethroid resistance locus. **A)** Genetic diversity (pi) across the 120kb *rp1* in Cameroon (orange), Malawi (blue), and Mozambique (green). **B)** Phylogeny of the ‘BAC25’ fragment (located 25kb along the BAC sequence and 9kb upstream of the *CYP6P9a* gene), which shows the most extreme difference in diversity between Cameroon (orange) and southern populations Malawi (blue) and Mozambique (green), which form a single clade. **C)** Phylogeny of the *CYP6P9a* gene sampled from throughout Africa, showing clear geographical divergence between southern Africa (Malawi and Mozambique; 100% bootstrap support), East Africa (Uganda; 87% bootstrap support) and West/Central Africa (Ghana, Benin, Cameroon; 78% bootstrap support). **D)** Haplotype network for Non-synonymous nucleotide variants in *CYP6P9a*. Light blue = haplotype shared between Malawi and Mozambique (MAL/MOZ); pink = haplotype shared between Benin, Cameroon and Ghana (BN/CAM/GH); teal = Benin (BN); red = Ghana (GH); orange = Cameroon (CAM); green = Mozambique (MOZ); blue = Malawi (MAL); purple = Uganda (UG). The size of the shapes is proportional to the frequency of the haplotype and numbers on each branch show the mutational steps separating haplotypes.

To assess the signature of a selective sweep associated with pyrethroid resistance, we carried out fine-scale polymorphism analysis across a 120kb genomic region spanning the *rp1* QTL in two highly pyrethroid resistant populations (Malawi and Mozambique) and a moderately resistant population (Cameroon). This revealed markedly reduced polymorphism in the vicinity of *CYP6P9a* (Fig 2A; S2A and S2B Fig), a key pyrethroid resistance gene, in Malawi and Mozambique but not in Cameroon. The greatest loss of diversity was closest to *CYP6P9a* at BAC25 (-9kb) (Fig 2A; S4 Table), which also produced geographical clustering based on an ML-tree (Fig 2B). The extent of this low polymorphism in Malawi is characterised by the complete absence of polymorphic sites at +61kb from *CYP6P9a* (BAC95), only 1 polymorphic site at +86kb, and 2 polymorphic sites at +36kb from *CYP6P9a*.

Analysis of the population from Cameroon shows that the relative diversity remains high across the *rp1* genomic fragment although a slight reduction is observed at *CYP6P9a* compared to other loci. Individual analysis of these loci shows that at -34kb from *CYP6P9a* (BAC 0) high haplotype diversity is noted. No predominant haplotype is observed with the most common haplotype, “CAM1”, having a frequency of 13.1% (5/38) (S2E Fig). A high proportion of haplotypes were singletons (16 out of 23) even in Malawi (7) and Mozambique (4). Additionally, there was a high number of mutational steps observed between haplotypes from each (>12) or between (>20) country (S2E Fig). A drastic change is observed closer to the *CYP6P9a* at -9kb (BAC25) where a strong signature of positive directional selection is observed in both Malawi and Mozambique. This reduced haplotype diversity is shown by the presence of a highly predominant haplotype MAL/MOZ1 only found in Malawi and Mozambique with a frequency of 92.8% (S3F Fig).

#### Maximum likelihood phylogenetic trees and haplotype network of *rp1*

Construction of Maximum likelihood (ML) phylogenetic trees of the five fragments further highlighted the reduced diversity close to *CYP6P9a* in the resistant populations of Malawi and Mozambique. The ML tree of the -34kb (BAC0) shows a high diversity of haplotypes for each of the three countries (S2C Fig), while the profile is very different at -9kb from *CYP6P9a* where all sequences from Mozambique and Malawi belong to a major haplotype while the Cameroon sample retained its high diversity (S2D Fig). Analysis of the TCS haplotype network confirms profiles obtained with the maximum likelihood phylogenetic trees for the 5 loci from -34kb to +86kb from *CYP6P9a* (S2E and S2F Fig). In general, Malawi and Mozambique formed their own clusters, with reduced genetic diversity shown by the fact that haplotypes from both countries had significantly fewer mutational steps between them (1–3 steps), while the more susceptible samples formed their own clusters with higher mutational steps (4–16 steps) for almost all of the BACs except for +86 (BAC 120).

#### Genetic diversity of the *CYP6P9a* pyrethroid resistance gene across Africa

To further confirm whether the selective sweep was driven by pyrethroid resistance across Africa, the full 1,965 bp of the *CYP6P9a* resistance gene was sequenced for a total of 59 mosquitoes from six countries (Benin, Cameroon, Ghana, Malawi, Mozambique and Uganda). Analysis of the genetic diversity of *CYP6P9a* revealed signatures of strong directional selection in Malawi and Mozambique but not in other regions (S5 Table; S3A Fig). In Malawi, only 5 polymorphic sites are observed (S5 Table; S3A Fig) and an AA indel in the 5’ UTR was found mainly in Southern Africa and not in the other countries. Nucleotide diversity (π) was lowest in Malawi (π = 0.0008) and Mozambique (0.0011), compared to Uganda (0.0015), Ghana (0.0020), Benin (0.0022) and Cameroon (0.0029) (S5 Table).

#### Maximum likelihood phylogenetic tree and haplotype network for *CYP6P9a*

Analysis of the ML tree of *CYP6P9a* further provided evidences of selection acting on this gene in southern Africa in contrast to other regions. *CYP6P9a* from Malawi and Mozambique formed a defined clade from the other four populations, dominated by a single haplotype (MAL/MOZ23-H25), which is the allele recently shown to exhibit the highest catalytic efficiency in metabolising pyrethroids (S3A Fig) [20]. Elsewhere, Benin and Ghana did not form a common clade despite their geographical proximity. Benin formed a cluster with Cameroon while Ghana was closer to Uganda, but they did not form a single cluster (Fig 2C; S3B Fig). All haplotypes from Uganda formed a cluster intermediate between Southern, West and Central African countries, which correlates well with the genetic structure obtained using microsatellite loci and reflects the geographical position of Uganda in East Africa, between Southern and West/Central Africa.

#### Genetic differentiation of the *CYP6P9a* gene

An index of genetic differentiation based on the nucleotide polymorphism (*K*_*ST*_*)* showed that the two Southern African populations of Malawi and Mozambique are genetically close (*K*_*ST*_ = 0.045; P<0.05). However, these highly pyrethroid resistant populations show extensive differentiation from all other populations, with *K*_*ST*_ estimates ranging from 0.347 (Malawi *vs*. Benin; P<0.001) to 0.574 (Mozambique *vs*. Uganda; P<0.001). The other four populations exhibit lower levels of genetic differentiation between them than seen between the two southern African populations, although in accordance to geographic proximity, the Benin samples are closer to Cameroon compared to the rest (S6 Table; S3D Fig). However, despite been geographically close to Benin in West Africa, the Ghana sample is more differentiated to Benin (*K*_*ST*_ = 0.50) than the Cameroon populations (*K*_*ST*_ = 0.13) in line with microsatellite results.

### 3-Assessing the role of insecticide-based interventions in the selective sweep in southern Africa

Due to the stronger signature of selection observed in the highly pyrethroid resistant populations of southern Africa, we aimed to establish if insecticide-based interventions were driving this selection. Mosquitoes collected in Malawi and Mozambique in 2002, predating the scaling up of malaria control interventions in southern Malawi and southern Mozambique were compared to mosquitoes collected in the same areas in 2009–2010 to determine whether the selective sweep coincided with the scale-up of pyrethroid-based vector control interventions that occurred over this period.

#### Microsatellite genetic diversity pre- and post-intervention

**Fig 3:**
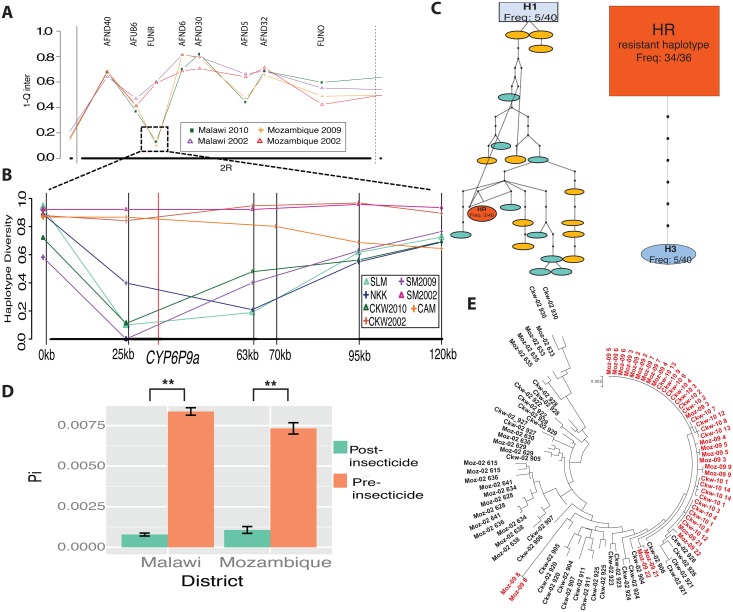
The selective sweep at *rp1* in southern Africa coincides with the scale-up in pyrethroid use in malaria control. **A)** Gene diversity at microsatellites on chromosome 2R in mosquitoes collected before widespread pyrethroid-based intervention in Malawi, 2002 (purple) and Mozambique, 2002 (red), and ‘post-intervention’ in Malawi, 2010 (green) and Mozambique, 2009 (yellow). **B)** Fine-scale nucleotide sequence analysis of the 120kb *rp1* locus in Cameroon (orange) or pre-intervention samples (Malawi (CKW2002) = brown and Mozambique (MOZ2009) = pink) compared to Post intervention samples from Chikwawa (green), Salima (light blue), Nkhotakota (blue) Malawi, Chokwe (purple) Mozambique, and Kaoma (dark blue) Zambia. **C)** TCS haplotype network at ‘BAC25’. Haplotypes including sequences from more than one location are denoted by an H and include the frequency, haplotypes from one location Malawi (blue) and Mozambique (yellow) are only present in pre-intervention samples. **D)** Change in average pairwise diversity (Pi) in the *CYP6P9a* gene pre- *vs*. post-intervention in Malawi and Mozambique. **E)** Phylogeny of *CYP6P9a* sequences including both Malawi (Ckw) and Mozambique (Moz) shows that post-intervention samples (red) are almost completely homogenous while pre-intervention samples (black) are diverse.

When the microsatellite diversity of pre- and post-intervention samples was compared, the only major variation occurs on the 2R chromosome, with higher genetic diversity in pre-intervention samples (H = 0.45) (Fig 3A). As already seen for the Africa-wide analysis, reduced diversity was detected on the 2R chromosomes around the *rp1* notably for the FunR, AFUB6 and AFND6, markers located close to the pyrethroid resistance gene. Per-locus gene diversity was compared among pre- and post-intervention samples. In both Malawi and Mozambique, the FunR locus showed a striking reduction in gene diversity between collection time-points, from 0.45 in 2002 to 0–0.15 in 2009–2011. The fact that this *rp1* region is the only area of the genome with a significant change of diversity level between pre and post-intervention samples further supports the recent occurrence of a selective sweep associated with implementation of vector control interventions.

#### Signature of selective sweep around 120kb genomic region of *rp1* in southern Africa

Fine-scale sequence analysis of polymorphism in the 120kb genomic region spanning the *rp1* locus revealed a loss of genetic diversity in the post-intervention samples occurring most strongly around the *CYP6P9a* resistance gene (S3B Fig; S4 Table). In contrast, samples collected from both Cameroon and pre-intervention in southern Africa showed no reduction of diversity across the entire *rp1* region (S4A and S4B Fig). Additional mosquitoes collected from across Malawi were sequenced and showed the selective sweep is consistent throughout southern Africa post-intervention (Fig 3B). A haplotype network of the closest fragment to *CYP6P9a*, BAC25 (-9kb), revealed the striking difference caused by control interventions. 22 unique haplotypes (with up to 23 mutational steps) were observed in pre-intervention samples, but only 2 in post-intervention samples (Fig 3C), which showed evidence of significant positive directional selection using both Tajima’s D (-2.10, -2.97) and Fu and Li’s D* (-2.94, -3.45) (S4 Table). The predominant post-intervention haplotype comprises 95% (68/72) of the sequences, highlighting the risk of a resistance allele becoming rapidly fixed over an 8 year period, from an original frequency of only 7.5%. Additionally, pre-intervention samples had a greater divergence and diversity based on a ML phylogenetic tree and higher numbers of singleton unique haplotypes, while post-intervention samples were nearly homogenous (S4C–S4F Fig).

#### Comparative analysis of *CYP6P9a* genetic diversity pre and post intervention

Analysis of the genetic diversity of *CYP6P9a* in pre- and post-intervention samples revealed this important pyrethroid resistance gene is under positive selection. Post-intervention samples show a signature of positive directional selection: negative Tajima’s D (-2.43, -2.54) and Fu and Li’s D* (-2.39, -2.25) (S5 Table); reduced pairwise diversity (Fig 3D) and few polymorphic sites (17 combined) (S5A and S5B Fig). In contrast, pre-intervention samples have 68 combined polymorphic sites, higher diversity, and no signature of selection. The ML tree revealed that pre-intervention samples are highly diverse in contrast to post-intervention samples, which are highly homogenous (Fig 3E). The contrast was also evident from the haplotype network, with a predominance of singleton haplotypes pre-intervention (82.5%) compared to only 7.5% post-intervention and the presence of a predominant haplotype in post-intervention samples (67.5%) corresponding to the resistance haplotype previously shown to be driving resistance in southern Africa (S6A Fig) [8]. When an Africa-wide comparison is performed for *CYP6P9a* polymorphisms, both pre-intervention samples are divergent and diverse while post-intervention samples form a cluster with low divergence (S6B Fig). Further analysis of the polymorphisms of the *CYP6P9a* coding region detected key amino acid changes shown to provide the highest catalytic efficiency when metabolising pyrethroids (S5B Fig) [20].

### 4-Whole genome sequencing validates the selective sweep at the *rp1* locus as the major genomic difference between pre- and post-intervention mosquitoes

**Fig 4:**
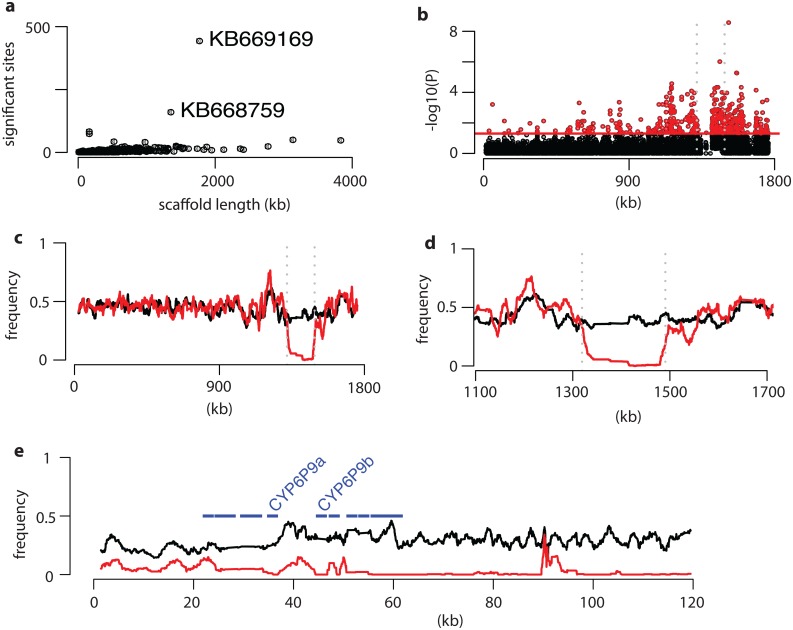
Genome-wide analysis of selection on the *An*. *funestus* genome. **A)** Sites with a significantly different allele frequency between 2002 and 2014 per genomic scaffold plotted against scaffold length identifies scaffold KB669169 as an outlier in which significantly skewed sites are over-represented. **B)** Plot of P-values of the difference in allele frequency for sites on scaffold KB669169. Grey dotted lines indicate the 120kb region represented by a sequenced BAC clone containing the *rp1* locus. **C)** Mean frequency of non-reference alleles (for 101 adjacent variant sites) on scaffold KB669169. **D)** Allele frequencies as in C, showing a magnified region spanning the *rp1* locus. **E)** Mean frequency of non-reference alleles (for 51 adjacent variant sites) on the BAC clone containing the *rp1* locus. Positions of genes in the P450 cluster are indicated and CYP6P9a and CYP6P9b are labelled.

Pooled template sequencing was carried out on two pools of mosquito genomic DNA: one from mosquitoes collected in 2014 and one from mosquitoes collected in 2002 in Malawi (S7 Table). Sequences were aligned to the FUMOZ reference genome (S8 Table) and stringent filtering performed to remove SNPs at the extremes of coverage depth (S9 Table). Analysis of a total of 979,808 variant sites genotyped in both samples detected 3,078 variant sites (on 368 genomic scaffolds) with significantly different allele frequencies between 2002 and 2014 collections. A significant correlation was observed between the number of significant sites plotted against total scaffold length (p<<0.01 for both Pearson’s and Spearman’s tests) (Fig 4A). However, a number of outlier scaffolds appear to be enriched for significant sites. The most extreme of these is scaffold KB669169, with more than 400 significant variants. This scaffold contains the *rp1* locus and most of the significant sites are clustered around this region (Fig 4B) with a striking loss of diversity between 2002 and 2014 evident across the locus (Fig 4C and 4D). This valley of reduced variability around *rp1* is the typical signature of selective sweeps as previously observed in other pool-seq studies [21, 22]. In no other scaffold was the signature so striking (S7 Fig), which validated the results of our microsatellite and targeted sequencing analyses. The *rp1* region of scaffold KB669169 contains a number of sequencing gaps. To ensure this did not affect our conclusions, sequence data were additionally aligned to the sequenced 120kb BAC clone containing *rp1* [7]. This confirmed the results observed in the whole genome analysis (Fig 4E; S8 Fig). The 2014 post-intervention sample exhibits a pattern similar to FUMOZ (the pyrethroid resistant laboratory colony) at *rp1* with reduced diversity spanning the cytochrome P450 cluster including the duplicated *CYP6P9a* and *CYP6P9b* genes, whereas the 2002 pre-intervention sample is highly diverse across the locus.

## Discussion

In order to help maintain the continued success of current insecticide-based malaria control interventions, this study has established the Africa-wide population structure and the full genomic signature of pyrethroid-based interventions in a major malaria-transmitting mosquito providing key evidence of the evolutionary response of mosquito populations to the massive scale up of insecticide-based interventions in Africa.

### 1-Patterns of genetic structure across *An*. *funestus* populations support the presence of barriers of gene flow

This study revealed that southern African populations of *An*. *funestus* are more genetically differentiated to other populations as they always form a unique cluster compared to other African regions based on both Bayesian analyses and *Fst* estimates. The population from Uganda appears to be intermediate between southern and West/Central Africa. This result is similar to patterns of genetic structure previously reported for this species [17]. Patterns of genetic structure observed in this study support the contrast in resistance patterns between populations of *An*. *funestus* and suggest the presence of barriers of gene flow between populations of this species. The causes of these barriers remain unknown although it could be associated with the absence of *An*. *funestus* around the Equatorial belt, or the presence of the Rift Valley which affects the population genetic structure of *An*. *gambiae* [23]. Such hypothesis will need to be validated by assessing more populations, e.g. from both sides of the Rift Valley. The patterns of gene flow described here for *An*. *funestus* give an indication on the risk and speed of spread of insecticide resistance alleles between these populations. This is further supported by the observation that the 119F resistance allele of the GSTe2 gene conferring DDT resistance probably arose in West or Central Africa, where it is common, but remains absent from southern Africa, despite selection pressure from DDT use [19].

### 2-Pyrethroid resistance is associated with a signature of positive directional selection

This study identified a major selective sweep on the 2R chromosomal arm, its location coinciding with that of the main pyrethroid-resistance QTL explaining 85% of genetic variance to resistance, and containing key cytochrome P450 genes conferring pyrethroid resistance [7, 24]. Overall, multiple analyses provide evidence for a selective sweep on the 2R chromosome driven by metabolic cytochrome P450-based pyrethroid resistance; (i) Reduced diversity of microsatellites flanking pyrethroid resistance genes on 2R in Malawi and Mozambique. (ii) Reduced genetic diversity of genomic sequences flanking pyrethroid resistance genes on 2R in Malawi and Mozambique. (iii) Reduced genetic diversity of the *CYP6P9a* gene in Malawi and Mozambique. (iv) No signature of a selective sweep prior to the widespread use of LLINs and IRS in Malawi and Mozambique.

#### (i) Reduced diversity of microsatellites flanking pyrethroid resistance genes on 2R

This study identified a valley of reduced diversity at the AFUB6 and FunR loci on the 2R chromosome, a typical signature of selective sweep similar to that described in malaria parasites in Southeast Asia in response to pyrimethamine treatment [25]. The reduced heterozygosity of these microsatellite markers and their position around the *rp1* pyrethroid resistance QTL point to a selective sweep associated with pyrethroid resistance. This is further supported by the lower diversity observed in the more resistant Southern populations of Malawi and Mozambique [4, 18, 26].

#### (ii) Reduced polymorphism of the 120kb *rp1* genomic region

Selection in the southern populations is confirmed by the continuous reduced genetic diversity for Malawi and Mozambique and also the reduced haplotype diversity in both countries. Selection is strongest around -9kb from *CYP6P9a* (BAC-25). This is due to its proximity to the two resistance genes, CYP6P9a and CYP6P9b. However, several other tests that can detect positive selection (MK, HKA, dN/dS and the Ka/Ks ratios) did not show any sign of positive selection. This can be explained by the fact that in a situation of near fixation of a selective sweep as seen in this study for the southern African populations, directional selection is rather indicated by reduced genetic variation [27]. Nevertheless, when estimating Ka/Ks ratios, a 2.64 and 2.5 fold reduction of the Ka/Ks estimates was observed in post intervention samples from Malawi and Mozambique respectively compared to the pre-intervention. The selection on the *rp1* region appears to be more extensive in Malawi than in Mozambique in line with previous reports [8]. In Malawi, the region under selection spans from -9kb from CYP6P9a and beyond +86kb, which reasonably could be above a region of 100kb. In Mozambique the region under selection spans from -9kb to +61kb, which is around 70kb.

#### (iii) Directional selection acting on *CYP6P9a* gene

The strong directional selection observed on *CYP6P9a* is only seen in southern Africa and correlates with the extensive pyrethroid resistance observed in this region with a high over-expression of this gene [4, 8, 18, 26]. The reduced *CYP6P9a* haplotype diversity with limited mutational steps between haplotypes in southern Africa is indicative of selection while in contrast the west and central African countries maintained high diversity. This lack of selection in other African regions could suggest that *rp1* may not be the main resistance region in other populations of *An*. *funestus* such as Uganda and Benin where pyrethroid resistance has been reported but with a less extreme over-expression of *CYP6P9a* [11, 13, 19].

The predominant haplotype in Malawi and Mozambique is a *CYP6P9a* allele recently shown to have greater efficiency in metabolising pyrethroids compared to susceptible alleles [8, 20]. Vector control interventions such as LLINs and IRS are largely implemented in these countries [4, 28]. This signature of directional selection on *CYP6P9a* is similar to the selection observed in the glutathione S-transferase *GSTe2* gene, another detoxification gene conferring resistance to DDT in *An*. *funestus* populations West/Central Africa [19]. Resistant samples from Benin exhibited a nearly fixed *GSTe2* haplotype in contrast to DDT susceptible populations where high genetic diversity is observed [19]. Evidence of a selective sweep around insecticide resistance genes has been reported in other species, such as *CYP6G1* in *D*. *melanogaster* [29] for which a single haplotype, containing a partial Accord transposable element in the 5’ UTR, confers DDT resistance [30].

Patterns of genetic differentiation based on *CYP6P9a* aligned with *F*_*ST*_ estimates obtained with microsatellites, where Malawi and Mozambique were significantly genetically differentiated from other African populations. The overall pattern of differentiation obtained here with *CYP6P9a* is also similar to that obtained with *GSTe2* gene [19] further supporting the presence of barriers to gene flow between southern Africa and other regions. Furthermore, the predominant *CYP6P9a* haplotype in southern Africa was completely absent from the other countries in line with the low level of gene flow observed between southern and West/Central Africa. This contrasting distribution of the *CYP6P9a* resistance haplotype is similar to the distribution of resistance mutations previously reported in *An*. *funestus* such as the L119F GSTe2 mutation, which is completely absent in Southern Africa [19]. However, one cannot exclude that the *CYP6P9a* resistant haplotype will spread to other regions with time as recently observed for the A296S RDL mutation in *An*. *funestus*. Indeed the *296S* resistant allele initially located only in West to East Africa and completely absent from southern Africa in 2010 [16] was recently detected in southern Africa, although at low frequency [15]. Similar geographical spread of resistance alleles over time has been observed for other resistance mutations such as knockdown resistance (*kdr*) mutations in mosquito species such as *An*. *gambiae* [31] or *Ae*. *aegypti* [32, 33].

#### (iv) Temporal analysis of southern African populations revealed that the scale-up of insecticide-based interventions is driving selection for insecticide resistance

In theory, causes other than insecticide use in public health could be selecting for insecticide resistance (e.g. agricultural pesticides or general pollution). However, while these may play a role, the scale-up of insecticide-based interventions across southern Africa was the only major influence that changed in such a short period of time (7–8 years). Based on the genetic diversity, haplotype networks, and the predicted maximum likelihood phylogenetic tree and the whole genome sequencing approach, the selective sweep observed around the *rp1* in general and particularly on *CYP6P9a* only happened after implementation of control interventions. Furthermore, the selection to near fixation of an allele highly efficient to metabolize pyrethroids [20] strengthens the hypothesis that this selective sweep was driven by these pyrethroid-based interventions. Such selection of a favourable allele is similar to previous examples of positive selection observed for target site mutations (ex. *kdr*) conferring resistance in many mosquito vectors [34–38] and selection linked to the *Ace-1* gene in *An*. *gambiae* [39]. In contrast to these examples, this is the first evidence of a signature of pyrethroid-based vector control such as LLINs on metabolic resistance mechanisms. The near fixation of a resistance haplotype over an 8 year period highlights the need to implement suitable resistance management early enough to prevent control failure. Selection due to pyrethroid interventions is especially alarming because currently pyrethroids are the only insecticide approved for use in bed nets.

## Conclusion

Altogether, this study provides conclusive evidence of the extensive selective sweep acting on cytochrome P450-based metabolic resistance to insecticides in mosquitoes. We conclude that positive selection on the region spanning the *rp1* pyrethroid resistance locus in *Anopheles funestus* has occurred in southern Africa between 2002 and 2009 in response to the increased use of pyrethroid-treated bednets. No major change in agricultural use of pyrethroids has occurred in southern Africa over this period, but LLIN use and, to a lesser extent pyrethroid-based IRS, has been scaled up massively in this period. This highlights the risk of relying on a single insecticide class for vector control and emphasizes the need for novel insecticides and vector control tools to tackle the spread of resistant vector populations.

## Methods

### Mosquito collections

*Anopheles funestus* mosquitoes were collected between 2009 and 2010 from the following locations: Chokwe, Mozambique (24° 33’S, 33° 01’E); Chikwawa, Malawi (16° 3’S, 34° 50’E); Tororo, Uganda (0° 45’N, 34° 5’E); Lagdo, Cameroon (9° 05’N, 13° 40’E); Pahou, Benin (6° 23'N, 2° 13'E); and Obuasi, Ghana (6° 12’N, 1° 40’W). Additional collections in southern Africa were made in 2011 in Salima (13° 57’S, 34° 30’E) and Nkhotakota (12° 56’S, 34° 17’E) in Malawi, plus collections from Chikwawa and Mozambique in 2002. Genomic DNA was extracted from whole mosquitoes using the method of Livak [40] or the DNeasy DNA Extraction Kit (Qiagen Inc., Valencia, CA, USA Mosquitoes were identified morphologically [41] and were species-typed using *An*. *funestus sensu stricto*-specific PCR primers [42]. Only confirmed *An*. *funestus s*.*s*. mosquitoes were used in this study.

Contrasting resistance profiles have been described for various populations of *An*. *funestus* across Africa. For example, the resistance pattern observed in North Cameroon in 2007 (DDT and dieldrin resistance)[16] is different to that of southern Africa (high pyrethroid, DDT and carbamate resistance)[4, 15], East Africa (pyrethroid and DDT resistance but full susceptibility to carbamates)[12] and Ghana (West Africa) (DDT resistance and pyrethroid resistance)[14].

### Microsatellite genotyping

Microsatellite markers were chosen to span the entire genome [17, 43]. Mosquitoes were collected from six countries (Obuasi, Ghana: N = 45, Pahou, Benin: N = 48, Lagdo, Cameroon: N = 48, Tororo, Uganda: N = 48) with two collection time points in Chikwawa, Malawi (2010: N = 48, 2002: N = 48) and Chokwe (2009: N = 48) and Morrumbene (2002: N = 45) Mozambique. 17 microsatellites (both di- and tri-nucleotide repeats) were amplified from genomic DNA using 1.5 μl of reaction Buffer, 0.2 μl of dNTP mix (25 mmol), 0.325 μl of both the forward (included a 19bp tag) and reverse primers, 0.2 μl of Hot Start Taq (Qiagen Inc.), 1 μl of MgCl_2_ and 1μl of genomic DNA (15ng/ul). Forward and reverse primers are listed in S1 Table. PCR thermocycler conditions were: 5min at 95°C followed by 35 cycles of denaturing at 94°C for 30s, annealing at 58°C for 30s and extension at 72°C for 30s, finishing with an extension step at 72°C for 10min. Fragment sizing was carried out using a Beckman Coulter CEQ8000. Fragment sizes were visualized and recorded using the fragment analysis software on the Beckman Coulter CEQ8000. Micro-Checker version 2.2.3 [44] was used to check for null allele and scoring errors.

### Population genetic analysis of microsatellites

Microsatellite data analysis was mainly carried out using Genepop version 4.0.10 [45]. Tests for deviation from Hardy Weinberg Equilibrium (HWE) were carried out for each locus using Genepop option 1.3. The null hypothesis was HWE and the alternative hypothesis a deficit of heterozygotes, and Bonferroni correction for multiple testing used to adjust the 0.05 and 0.01 critical values. The inbreeding coefficient *F*_*IS*_, linkage disequilibrium (LD), log likelihood ratio statistics (G-test) and tables created using Markov chain algorithm of Raymond & Rousset [45] were all preformed. Gene diversity at each microsatellite locus, estimated by 1-Q_(inter)_ where Q_(inter)_ is the homozygosity among individuals, and among populations. Genetic differentiation (*F*_*ST*_) were estimated using Genepop [45]. Pairwise *F*_*st*_ values were used to generate neighbour joining trees using the software MEGA 5.2 [46].

### Bayesian analysis of population structure

Bayesian analysis of population structure was implemented using STRUCTURE version 2.3.4 [47]. Individually based admixture models were used to estimate the ancestral allele source observed in each individual, where the ancestral source population is unknown. A total of 285 individuals from six African countries, genotyped at 16 loci, were analysed for cluster number K = 1–12 (10 replicate runs for each) using a set of 9 putatively neutral loci and comparing 8 2R loci to the 8 non-2R markers. A burn-in period of 50,000 generations and Markov Chain Monte Carlo (MCMC) simulations of 100,000 iterations were used. The admixture model was used as it allows individuals to have mixed ancestry where a fraction (qk) of the genome of an individual comes from an ancestral cluster (where tkqk = 1)[47]. Structure Harvester [48] was used to infer the most likely number of clusters (K) using Evanno’s method [49]. CLUMPP [50] was used to collate the data from all 10 runs for each given K value, for plotting.

### Analysis of polymorphism in the pyrethroid resistance *rp1* QTL and the *CYP6P9a* gene

Five DNA fragments evenly spaced to span the 120kb BAC, originally isolated using a laboratory colony, FUMOZ [7], upstream and downstream of *CYP6P9a* were sequenced in order to assess the presence of a selective sweep around this key resistance gene across Africa (Cameroon, Malawi and Mozambique). Primers used to amplify the fragments are listed in S1 Table.

A subset of 10 mosquitoes used for the microsatellite analysis were randomly selected from each collection site for analysis. For BAC fragments, 770-1000bp were sequenced using: 3 μl of 10X KAPA Taq buffer A (KAPA Biosystems), 0.24 μl of 5 U/μl KAPA Taq, 0.24 μl of 25 μM dNTP mix, 1.5 μl of 25μM MgCl_2_, 1.02 μl each of antisense and sense primers, 2 μl of gDNA (10ng), and 22.48 μl of dH_2_O. The 30 μl solution underwent a denaturing step at 95°C for 5min, followed by 35 cycles of 94°C for 30s, 57°C for 30s and 72°C for 1min and 30s, followed by a final extension step of 72°C for 10min.

The *CYP6P9a* gene was amplified from the same gDNA samples used for the *rp1* BAC analysis. *CYP6P9a* was amplified, covering the 5’ UTR, the gene’s two exons and one intron with a total sequence of approximately 2kb using previously published primers and parameters [8]. PCR products were purified using the QIAquick PCR Purification Kit and directly sequenced using Sanger sequencing. Sequences were first analysed for quality then manually assessed for polymorphisms using BioEdit [51]. Sequences were aligned using ClustalW [52].

Heterozygous sites in sequence data were phased using the PHASER algorithm implemented in DnaSP version 5.10 [53]. For all sequences, DnaSP was used to calculate the number of segregating sites (S), the number of haplotypes (h), the nucleotide diversity (π) and the haplotype diversity (Hd). Two tests of neutrality, Tajima’s D and Fu and Li’s D*, were also carried out. For sequences encoding proteins, 1524bp of the *CYP6P9a* gene, the numbers of synonymous and nonsynonymous polymorphisms and nonsynonymous (K_A_) and synonymous (K_S_) polymorphisms per site were calculated. The *K*_*ST*_ statistic in dnaSP 5.1 [54] was used to estimate the levels of pair-wise genetic differentiation between populations. The statistical significance of the *K*_ST_* estimates was assessed by permutation of subpopulations identities and re-calculating *K*_ST_* 10,000 times as implemented in dnaSP5.1.

Hudson, Kreitman and Aguade’s (HKA) and McDonald and Kreitman’s (MK) tests of neutrality were performed using the *An*. *gambiae* orthologue of *CYP6P9a*, *CYP6P3* (AGAP002865-PA) as an out-group. For the MK test on the BAC25 fragment, which spans the CYP6AA2 gene in *An*. *funestus*, the *An*. *gambiae CYP6AA2* gene (AGAP002862-PA) was used as an out-group.

Maximum likelihood phylogenetic trees where generated for the BAC25 sequences (Tamura’s Model) and for *CYP6P9a* (Kimura’s 2-parameter model) using MEGA 5.2, with 500 bootstrap replicates [46]. Haplotype networks were determined, based on a 95% connection limit with gaps treated as a fifth state, using TCS [55]. Individual haplotypes were labelled by colour and shape (circle denoting haplotypes unique to only one sequence, squares denote haplotypes containing multiple sequences).

### Whole genome sequencing-based scan of selective sweep in pooled mosquitoes pre- (2002) and post-intervention (2014)

A whole genome scan was performed comparatively between pre- and post-intervention mosquitoes in order to detect all selective sweep signatures associated with the scale-up of insecticide-based interventions. Pooled template whole genome sequencing libraries were generated as follows. Genomic DNA was purified from individual female mosquitoes collected from Chikwawa in Malawi in 2014 and 2002 using the DNeasy Blood and Tissue Kit (QIAgen, Hilden, Germany), following the manufacturer’s instructions and including an RNase treatment step to remove RNA. The gDNA from each mosquito was quantified using the Quant-iT PicoGreen dsDNA Assay Kit (Thermo-Fisher, Waltham, USA) on a FLUOstar Omega Microplate Reader (BMG Labtech, Aylesbury, UK). Equal quantities of gDNA from 40 individuals for each collection were pooled in equal amounts and the pools used to generate Illumina TruSeq Nano DNA fragment libraries (Illumina, San Diego, USA) with an insert size of 350bp. Libraries were sequenced (2x125bp paired-end sequencing) on different lanes of an Illumina HiSeq2500 (Illumina) at the Center for Genomics Research (University of Liverpool, UK), each multiplexed with three other libraries (not used in this study), to produce approximately 50,000,000 read pairs per library.

Raw sequence reads were trimmed of adapter sequence and low quality bases, using cutadapt [56] and sickle [57], and filtered to remove trimmed reads shorter than 10bp. Trimmed reads were aligned to the *Anopheles funestus* (FUMOZ) reference genome sequence (version Afun1.3) downloaded from VectorBase [58], using bowtie2 [59]. Aligned reads were filtered to remove duplicate reads and those not properly paired (mapped in forward and reverse orientation within 500bp of each other) or with mapping quality <10. Retained reads were used to detect single nucleotide polymorphisms (SNP).

SNP calling was carried out using SNVer [60]. SNPs were filtered to remove those called in regions of in the top or bottom 25% of read coverage depth, to remove artefacts caused by misaligned paralogous sequences. Variant sites with coverage data within the allowed range for both libraries were analysed to identify those with significantly different allele frequencies in each pool. A chi-squared test for a significant difference in allele frequency was applied to each variant site. P-values were corrected for multiple testing using the method of Benjamini and Hochberg [61] and sites with an adjusted p-value less than 0.05 were considered significant. In addition, a rolling mean non-reference allele frequency was calculated and plotted for sets of 101 adjacent sites incremented by one site per step across each scaffold.

### Data access

The DNA sequences reported in this paper have been deposited in the GenBank database accession numbers: KU168962-199123. The whole genome sequence read data reported in this study were submitted to the European Nucleotide Archive (ENA) under the study accession PRJEB13485 (http://www.ebi.ac.uk/ena/data/view/PRJEB13485) and the sample accessions ERS1115465 and ERS1115466.

## Supporting Information

S1 Fig African-wide population structure. **(A)** Neighbor-joining tree based on the F_st_ of 8 microsatellites on 2R. **(B)** Neighbor-joining tree based on the F_st_ of 8 microsatellites non 2R chromosome markers. **(C)** Bayesian population structure of Africa based on 8 microsatellites spanning rp1 QTL on 2R chromosome: BEN-Benin, CMR = Cameroon, GHA = Ghana, MWI = Malawi, MOZ = Mozambique, UGA = Uganda. **(D)** Bayesian population structure of other 8 microsatellites from other chromosomes apart 2R, in order to assess how the rp1 markers are skewing the population structure.
(TIFF)

S2 Fig **Analysis of the rp1 QTL** BAC loci 25 **(A)** and BAC 70 **(B)** across the more resistant population (MAL—Malawi and MOZ—Mozambique) and the more susceptible samples (CAM—Cameroon). The polymorphic positions are indicated with and the second numbers (n) indicates the haplotype frequency. **(C)** Maximum likelihood tree of fragment at -34kb of CYP6P9a (BAC0) and **(D)** is for the fragment at -9kb of CYP6P9a (BAC 25). **(E)** is the haplotype network of BAC0 (-34kb) and **(F)** is for BAC25 (-9kb) where pink represents haplotype dominant in both Malawi and Mozambique. The size of the polygon reflects the frequency of the haplotype. Segregating mutation is represented by each node and polymorphic positions are given above the branches.
(TIFF)

S3 Fig Africa-wide 6P9a diversity. **(A)** Haplotype distribution of non-synonymous equivalent of amino acid protein variants. Highlighted in red are the amino acid changes linked to pyrethroid resistance [20]. **(B)** Maximum likelihood tree of the *CYP6P9a* gene (non-synonymous) changes) for six samples (BN—Benin, CAM—Cameroon, GH—Ghana, MAL—Malawi, MOZ—Mozambique and UG—Uganda). **(C)** Haplotype network of *CYP6P9a* for individual countries for coding region. The size of the polygon reflects the frequency of the haplotype and colour represents the countries (BN (Benin)–Blue, CAM (Cameroon)–Yellow, GH (Ghana)–Red, MAL (Malawi)—Grey, MOZ (Mozambique)—Green and UG (Uganda)—Purple). Segregating mutation is represented by each node and rectangular boxes represent major haplotype. **(D)** Neighbour joining tree based on genetic distances from *K*_*ST*_ estimates of pairwise population comparison.
(TIFF)

S4 Fig Analysis of the *rp1* BAC clones showing higher selection near *CYP6P9a*. Analysis of two BAC sequences from southern Africa BAC0 on the 5’UTR and BAC25 near the gene CYP6P9a. **(A-B)**, Haplotypes from post-intervention samples (red) and pre-intervention from Malawi (blue), and Mozambique (green) including the frequency in brackets and the SNP location on the top x-axis. **(C-D)**, ML-tree of pre-intervention (black) and post-intervention (red) show more divergence at BAC0 while BAC25 shows distinct grouping between pre and post. **(E-F)**, Haplotype network where the size correlated to frequency. The red denotes samples collected post-intervention, blue were collected pre-intervention and green contains sequences from both time points.
(TIFF)

S5 Fig Loss of diversity post—intervention in the gene CYP6P9a. **(A)** All SNPs in pre-intervention samples (blue and green) and post-intervention (red) compared to a susceptible lab strain, Fang (black) including frequency in brackets and the position on the top x-axis. **(B)** Haplotypes based on amino acid changes pre (blue and green) versus post-intervention (red) shows a major loss of diversity. SNPs highlighted in yellow denote changes implicated in increased catalytic function [20].
(TIFF)

S6 Fig Impact of control interventions on genetic diversity of CYP6P9a. **(A)** The TCS haplotype network of pre- versus post-intervention samples based on the coding region including haplotypes with more than one sequence (red) and singular haplotypes from Malawi (orange) and Mozambique (blue). **(B)** Africa-wide Neighbor-Joining tree of CYP6P9a shows geographical clustering where southern Africa (blue and orange) is divergent. Within the southern Africa cluster there is a lack of diversity in Mozambique (MOZ 2009) and Malawi (MWI 2010).
(TIFF)

S7 Fig Genome-wide analysis of selection on the *An*. *funestus* genome showing other scaffolds with sites with a significantly different allele frequency between 2002 and 2014. These show no striking valley of reduced variability in contrast to KB669169 spanning *rp1*. **(A)** Plot of P-values of the difference in allele frequency for sites on respective scaffold. **(B)** Mean frequency of non-reference alleles (for 101 sites) on respective scaffold. The black line is for MWI-2002 and the red line for MWI-2014.
(TIFF)

S8 Fig Genomic location of the selective sweep. **(A)** Gene annotation of the rp1 region view from the Vectobase screenshot. The region from approximately 1.32 to 1.48 Mb corresponds to the sequence BAC containing rp1. Blue bars represent scaffolded contigs and spaces are unsequenced assembly gaps, which are common in this region. Red boxes represent annotated genes. **(B)** Contrasting polymorphism patterns between pre- and post-intervention samples: Data aligned to BAC (IGV screenshot). Full-length (120kb) BAC sequence. The top track shows the position on the BAC, the second and third show alignment depth (on a log scale for display purposes) for 2002 and 2014, respectively (coverage depth is capped at >100x). Grey columns represent bases identical to the reference sequence while coloured columns indicate variant sites with a minor allele frequency >10%. The fourth track shows genes of the P450 cluster.
(TIFF)

S1 Table Polymorphism at 16 microsatellite loci in *An*. *funestus* collected from six African countries. (PDF)

S2 Table Genetic Differentiation between six African populations of *An*. *funestus*. (PDF)

S3 Table Bayesian assignment of six African populations of *An*. *funestus*. (PDF)

S4 Table Genetic diversity parameters for 5 DNA sequences spanning the 120kb *rp1* QTL genomic region. (PDF)

S5 Table Genetic parameters for *CYP6P9a* across Africa and between pre- and post-intervention samples. (PDF)

S6 Table Genetic differentiation (*K*_*ST*_) based on polymorphism in the CYP6P9a gene. (PDF)

S7 Table Descriptive statistics of POOLseq sequence read data for field-caught mosquitoes from Malawi. (PDF)

S8 Table Descriptive statistics of POOLseq sequence alignments and coverage depth of field-caught mosquitoes from Malawi. (PDF)

S9 Table Numbers of SNPs before and after filtering for coverage depth for POOLseq sequence alignments of field-caught mosquitoes from Malawi, individually and both together. (PDF)

S10 Table Microsatellite marker information and BAC clone primer information. (PDF)

S1 Text Additional methods and results. (DOCX)

## References

[1] Bhatt S, Weiss DJ, Cameron E, Bisanzio D, Mappin B, Dalrymple U, et al The effect of malaria control on Plasmodium falciparum in Africa between 2000 and 2015. Nature. 2015;526(7572):207–11. 10.1038/nature15535 26375008 PMC4820050

[2] WHO. Global Plan for Insecticide Resistance Management (GPIRM). Programme WGM, editor. Geneva, Switzerland: World Health Organization; 2012.

[3] Hemingway J, Vontas J, Poupardin R, Raman J, Lines J, Schwabe C, et al Country-level operational implementation of the Global Plan for Insecticide Resistance Management. Proc Natl Acad Sci U S A. 2013;110(23):9397–402. 10.1073/pnas.1307656110 23696658 PMC3677419

[4] Wondji CS, Coleman M, Kleinschmidt I, Mzilahowa T, Irving H, Ndula M, et al Impact of pyrethroid resistance on operational malaria control in Malawi. Proc Natl Acad Sci U S A. 2012;109(47):19063–70. Epub 2012/11/03. 10.1073/pnas.1217229109 23118337 PMC3511128

[5] Clarkson CS, Weetman D, Essandoh J, Yawson AE, Maslen G, Manske M, et al Adaptive introgression between Anopheles sibling species eliminates a major genomic island but not reproductive isolation. Nat Commun. 2014;5:4248 10.1038/ncomms5248 24963649 PMC4086683

[6] Pinto J, Lynd A, Vicente JL, Santolamazza F, Randle NP, Gentile G, et al Multiple origins of knockdown resistance mutations in the Afrotropical mosquito vector Anopheles gambiae. PLoS One. 2007;2(11):e1243 Epub 2007/11/29. 10.1371/journal.pone.0001243 18043750 PMC2080755

[7] Wondji CS, Irving H, Morgan J, Lobo NF, Collins FH, Hunt RH, et al Two duplicated P450 genes are associated with pyrethroid resistance in Anopheles funestus, a major malaria vector. Genome Res. 2009;19(3):452–9. Epub 2009/02/07. 10.1101/gr.087916.108 19196725 PMC2661802

[8] Riveron JM, Irving H, Ndula M, Barnes KG, Ibrahim SS, Paine MJ, et al Directionally selected cytochrome P450 alleles are driving the spread of pyrethroid resistance in the major malaria vector Anopheles funestus. Proc Natl Acad Sci U S A. 2013;110(1):252–7. Epub 2012/12/19. 10.1073/pnas.1216705110 23248325 PMC3538203

[9] Choi KS, Christian R, Nardini L, Wood OR, Agubuzo E, Muleba M, et al Insecticide resistance and role in malaria transmission of Anopheles funestus populations from Zambia and Zimbabwe. Parasit Vectors. 2014;7:464 10.1186/s13071-014-0464-z 25293669 PMC4197278

[10] Coetzee M, Koekemoer LL. Molecular systematics and insecticide resistance in the major African malaria vector Anopheles funestus. Annu Rev Entomol. 2013;58:393–412. 10.1146/annurev-ento-120811-153628 23317045

[11] Djouaka R, Irving H, Tukur Z, Wondji CS. Exploring Mechanisms of Multiple Insecticide Resistance in a Population of the Malaria Vector Anopheles funestus in Benin. PLoS One. 2011;6(11):e27760 Epub 2011/11/24. 10.1371/journal.pone.0027760 22110757 PMC3218031

[12] Morgan JC, Irving H, Okedi LM, Steven A, Wondji CS. Pyrethroid resistance in an Anopheles funestus population from Uganda. PLoS One. 2010;5(7):e11872 Epub 2010/08/06. 10.1371/journal.pone.0011872 20686697 PMC2912372

[13] Mulamba C, Riveron JM, Ibrahim SS, Irving H, Barnes KG, Mukwaya LG, et al Widespread pyrethroid and DDT resistance in the major malaria vector Anopheles funestus in East Africa is driven by metabolic resistance mechanisms. PLoS One. 2014;9(10):e110058 10.1371/journal.pone.0110058 25333491 PMC4198208

[14] Okoye PN, Brooke BD, Koekemoer LL, Hunt RH, Coetzee M. Characterisation of DDT, pyrethroid and carbamate resistance in Anopheles funestus from Obuasi, Ghana. Trans R Soc Trop Med Hyg. 2008;102(6):591–8. Epub 2008/04/15. 10.1016/j.trstmh.2008.02.022 18405930

[15] Riveron JM, Chiumia M, Menze BD, Barnes KG, Irving H, Ibrahim SS, et al Rise of multiple insecticide resistance in Anopheles funestus in Malawi: a major concern for malaria vector control. Malar J. 2015;14:344 10.1186/s12936-015-0877-y 26370361 PMC4570681

[16] Wondji CS, Dabire RK, Tukur Z, Irving H, Djouaka R, Morgan JC. Identification and distribution of a GABA receptor mutation conferring dieldrin resistance in the malaria vector Anopheles funestus in Africa. Insect Biochem Mol Biol. 2011;41(7):484–91. Epub 2011/04/20. 10.1016/j.ibmb.2011.03.012 21501685 PMC3579012

[17] Michel AP, Ingrasci MJ, Schemerhorn BJ, Kern M, Le Goff G, Coetzee M, et al Rangewide population genetic structure of the African malaria vector Anopheles funestus. Mol Ecol. 2005;14(14):4235–48. 10.1111/j.1365-294X.2005.02754.x 16313589

[18] Cuamba N, Morgan JC, Irving H, Steven A, Wondji CS. High level of pyrethroid resistance in an Anopheles funestus population of the Chokwe District in Mozambique. PLoS One. 2010;5(6):e11010 Epub 2010/06/15. 10.1371/journal.pone.0011010 20544036 PMC2882342

[19] Riveron JM, Yunta C, Ibrahim SS, Djouaka R, Irving H, Menze BD, et al A single mutation in the GSTe2 gene allows tracking of metabolically-based insecticide resistance in a major malaria vector. Genome Biol. 2014;15(2):R27 10.1186/gb-2014-15-2-r27 24565444 PMC4054843

[20] Ibrahim SS, Riveron JM, Bibby J, Irving H, Yunta C, Paine MJ, et al Allelic Variation of Cytochrome P450s Drives Resistance to Bednet Insecticides in a Major Malaria Vector. PLoS Genet. 2015;11(10):e1005618 10.1371/journal.pgen.1005618 26517127 PMC4627800

[21] Rubin CJ, Zody MC, Eriksson J, Meadows JR, Sherwood E, Webster MT, et al Whole-genome resequencing reveals loci under selection during chicken domestication. Nature. 2010;464(7288):587–91. 10.1038/nature08832 20220755

[22] Schlotterer C, Tobler R, Kofler R, Nolte V. Sequencing pools of individuals—mining genome-wide polymorphism data without big funding. Nat Rev Genet. 2014;15(11):749–63. 10.1038/nrg3803 25246196

[23] Lehmann T, Hawley WA, Grebert H, Danga M, Atieli F, Collins FH. The Rift Valley complex as a barrier to gene flow for Anopheles gambiae in Kenya. J Hered. 1999;90(6):613–21. 10589511 10.1093/jhered/90.6.613

[24] Wondji CS, Morgan JC, Coetzee M, Hunt R, Steen K, Black WC, et al Mapping a Quantitative Trait Locus conferring pyrethroid resistance in the African malaria vector Anopheles funestus. BMC Genomics. 2007;8: 34 10.1186/1471-2164-8-34 17261170 PMC1790900

[25] Nair S, Williams JT, Brockman A, Paiphun L, Mayxay M, Newton PN, et al A selective sweep driven by pyrimethamine treatment in southeast asian malaria parasites. Molecular Biology and Evolution. 2003;20(9):1526–36. 10.1093/molbev/msg162 12832643

[26] Brooke BD, Kloke G, Hunt RH, Koekemoer LL, Temu EA, Taylor ME, et al Bioassay and biochemical analyses of insecticide resistance in southern African Anopheles funestus (Diptera: Culicidae). Bull Entomol Res. 2001;91(4):265–72. 11587622 10.1079/ber2001108

[27] Biswas S, Akey JM. Genomic insights into positive selection. Trends Genet. 2006;22(8):437–46. Epub 2006/07/01. 10.1016/j.tig.2006.06.005 16808986

[28] Thomsen EK, Strode C, Hemmings K, Hughes AJ, Chanda E, Musapa M, et al Underpinning sustainable vector control through informed insecticide resistance management. PLoS One. 2014;9(6):e99822 10.1371/journal.pone.0099822 24932861 PMC4059741

[29] Schlenke TA, Begun DJ. Strong selective sweep associated with a transposon insertion in Drosophila simulans. Proc Natl Acad Sci U S A. 2004;101(6):1626–31. Epub 2004/01/28. 10.1073/pnas.0303793101 14745026 PMC341797

[30] Daborn PJ, Yen JL, Bogwitz MR, Le Goff G, Feil E, Jeffers S, et al A single p450 allele associated with insecticide resistance in Drosophila. Science. 2002;297(5590):2253–6. 10.1126/science.1074170 12351787

[31] Ranson H, N'Guessan R, Lines J, Moiroux N, Nkuni Z, Corbel V. Pyrethroid resistance in African anopheline mosquitoes: what are the implications for malaria control? Trends Parasitol. 2011;27(2):91–8. Epub 2010/09/17. 10.1016/j.pt.2010.08.004 20843745

[32] Ishak IH, Jaal Z, Ranson H, Wondji CS. Contrasting patterns of insecticide resistance and knockdown resistance (kdr) in the dengue vectors Aedes aegypti and Aedes albopictus from Malaysia. Parasit Vectors. 2015;8:181 10.1186/s13071-015-0797-2 25888775 PMC4377062

[33] Vontas J, Kioulos E, Pavlidi N, Morou E, della Torre A, Ranson H. Insecticide resistance in the major dengue vectors Aedes albopictus and Aedes aegypti. Pesticide Biochemistry and Physiology. 2012;104(2):126–31.

[34] Lumjuan N, Rajatileka S, Changsom D, Wicheer J, Leelapat P, Prapanthadara LA, et al The role of the *Aedes aegypti* Epsilon glutathione transferases in conferring resistance to DDT and pyrethroid insecticides. Insect Biochemistry and Molecular Biology. 2011;41(3):203–9. 10.1016/j.ibmb.2010.12.005 21195177

[35] Riveron JM, Yunta C, Ibrahim SS, Djouaka R, Irving H, Menze BD, et al A single mutation in the GSTe2 gene allows tracking of metabolically based insecticide resistance in a major malaria vector. Genome Biol. 2014;15(2). 10.1186/gb-2014-15-2-r27 PMC4054843 24565444

[36] Williamson MS, Denholm I, Bell CA, Devonshire AL. Knockdown resistance (kdr) to DDT and pyrethroid insecticides maps to a sodium channel gene locus in the housefly (Musca domestica). Mol Gen Genet. 1993;240(1):17–22. Epub 1993/07/01. 8101963 10.1007/BF00276878

[37] Wondji CS, Dabire RK, Tukur Z, Irving H, Djouaka R, Morgan JC. Identification and distribution of a GABA receptor mutation conferring dieldrin resistance in the malaria vector Anopheles funestus in Africa. Insect Biochem Molec. 2011;41(7):484–91. 10.1016/j.ibmb.2011.03.012 PMC3579012 21501685

[38] Wondji CS, De Silva WAPP, Hemingway J, Ranson H, Karunaratne SHPP. Characterization of knockdown resistance in DDT- and pyrethroid-resistant Culex quinquefasciatus populations from Sri Lanka. Trop Med Int Health. 2008;13(4):548–55. 10.1111/j.1365-3156.2008.02033.x 18312471

[39] Weetman D, Mitchell SN, Wilding CS, Birks DP, Yawson AE, Essandoh J, et al Contemporary evolution of resistance at the major insecticide target site gene Ace-1 by mutation and copy number variation in the malaria mosquito Anopheles gambiae. Mol Ecol. 2015;24(11):2656–72. Epub 2015/04/14. 10.1111/mec.13197 25865270 PMC4447564

[40] Livak KJ. Organization and mapping of a sequence on the Drosophila melanogaster X and Y chromosomes that is transcribed during spermatogenesis. Genetics. 1984;107(4):611–34. Epub 1984/08/01. 6430749 10.1093/genetics/107.4.611 PMC1202380

[41] Gillies MT, Coetzee M. A supplement to the Anophelinae of Africa south of the Sahara (Afrotropical region). Johannesburg: South African Institute for medical research; 1987 143 p.

[42] Koekemoer LL, Kamau L, Hunt RH, Coetzee M. A cocktail polymerase chain reaction assay to identify members of the Anopheles funestus (Diptera: Culicidae) group. Am J Trop Med Hyg. 2002;66(6):804–11. 12224596 10.4269/ajtmh.2002.66.804

[43] Cohuet A, Dia I, Simard F, Raymond M, Fontenille D. Population structure of the malaria vector Anopheles funestus in Senegal based on microsatellite and cytogenetic data. Insect Mol Biol. 2004;13(3):251–8. 10.1111/j.0962-1075.2004.00482.x 15157226

[44] van Oosterhout C., Hutchinson W.F., Wills D.P.M., P. S. MICRO-CHECKER: software for identifying and correcting genotyping errors in microsatellite data. Molecular Ecology Notes. 2004;4:535–8

[45] Rousset F. genepop'007: a complete re-implementation of the genepop software for Windows and Linux. Mol Ecol Resour. 2008;8(1):103–6. 10.1111/j.1471-8286.2007.01931.x 21585727

[46] Tamura K, Peterson D, Peterson N, Stecher G, Nei M, Kumar S. MEGA5: molecular evolutionary genetics analysis using maximum likelihood, evolutionary distance, and maximum parsimony methods. Mol Biol Evol. 2011;28(10):2731–9. Epub 2011/05/07. 10.1093/molbev/msr121 21546353 PMC3203626

[47] Pritchard JK, Stephens M, Donnelly P. Inference of population structure using multilocus genotype data. Genetics. 2000;155(2):945–59. 10835412 10.1093/genetics/155.2.945 PMC1461096

[48] Earl D, vonHoldt B. STRUCTURE HARVESTER: a website and program forvisualizing STRUCTURE output and implementing the Evanno method. Conservation Genetics Resources. 2012;4:359–61.

[49] Evanno G, Regnaut S, Goudet J. Detecting the number of clusters of individuals using the software STRUCTURE: a simulation study. Mol Ecol. 2005;14(8):2611–20. 10.1111/j.1365-294X.2005.02553.x 15969739

[50] Jakobsson M, Rosenberg NA. CLUMPP: a cluster matching and permutation program for dealing with label switching and multimodality in analysis of population structure. Bioinformatics. 2007;23(14):1801–6. 10.1093/bioinformatics/btm233 17485429

[51] Hall TA. BioEdit: a user-friendly biological sequence alignment editor and analysis program for Windows 95/98/NT. Nucleic Acids Symposium Series. 1999:95–8.10780396

[52] Thompson JD, Higgins DG, Gibson TJ. CLUSTAL W: improving the sensitivity of progressive multiple sequence alignment through sequence weighting, position-specific gap penalties and weight matrix choice. Nucleic Acids Res. 1994;22(22):4673–80. 7984417 10.1093/nar/22.22.4673 PMC308517

[53] Librado P, Rozas J. DnaSP v5: a software for comprehensive analysis of DNA polymorphism data. Bioinformatics. 2009;25(11):1451–2. Epub 2009/04/07. 10.1093/bioinformatics/btp187 19346325

[54] Hudson RR, Slatkin M, Maddison WP. Estimation of levels of gene flow from DNA sequence data. Genetics. 1992;132(2):583–9. 1427045 10.1093/genetics/132.2.583 PMC1205159

[55] Clement M, Posada D, Crandall KA. TCS: a computer program to estimate gene genealogies. Mol Ecol. 2000;9(10):1657–9. 11050560 10.1046/j.1365-294x.2000.01020.x

[56] Martin M. Cutadapt removes adapter sequences from high-throughput sequencing reads. EMBnet. 2011;17:10–2.

[57] Joshi NA, Fass JN. Sickle: A sliding-window, adaptive, quality-based trimming tool for FastQ files (Version 1.33) [Software]. https://githubcom/najoshi/sickle. 2011.

[58] Giraldo-Calderon GI, Emrich SJ, MacCallum RM, Maslen G, Dialynas E, Topalis P, et al VectorBase: an updated bioinformatics resource for invertebrate vectors and other organisms related with human diseases. Nucleic Acids Res. 2015;43(Database issue):D707–13. 10.1093/nar/gku1117 25510499 PMC4383932

[59] Langmead B, Salzberg SL. Fast gapped-read alignment with Bowtie 2. Nat Methods. 2012;9(4):357–9. 10.1038/nmeth.1923 22388286 PMC3322381

[60] Wei Z, Wang W, Hu P, Lyon GJ, Hakonarson H. SNVer: a statistical tool for variant calling in analysis of pooled or individual next-generation sequencing data. Nucleic Acids Res. 2011;39(19):e132 10.1093/nar/gkr599 21813454 PMC3201884

[61] Benjamini Y, Hochberg Y. Controlling the False Discovery Rate: A Practical and Powerful Approach to Multiple Testing. Journal of the Royal Statistical Society Series B (Methodological) 1995;57(1):289–300

