# Supplementary figures and images for: Genomic Footprints of Selective Sweeps from Metabolic Resistance to Pyrethroids in African Malaria Vectors Are Driven by Scale up of Insecticide-Based Vector Control
Source: PLoS Genet. 2017 Feb 2;13(2):e1006539. doi: 10.1371/journal.pgen.1006539 (PMC5289422; doi:10.1371/journal.pgen.1006539)

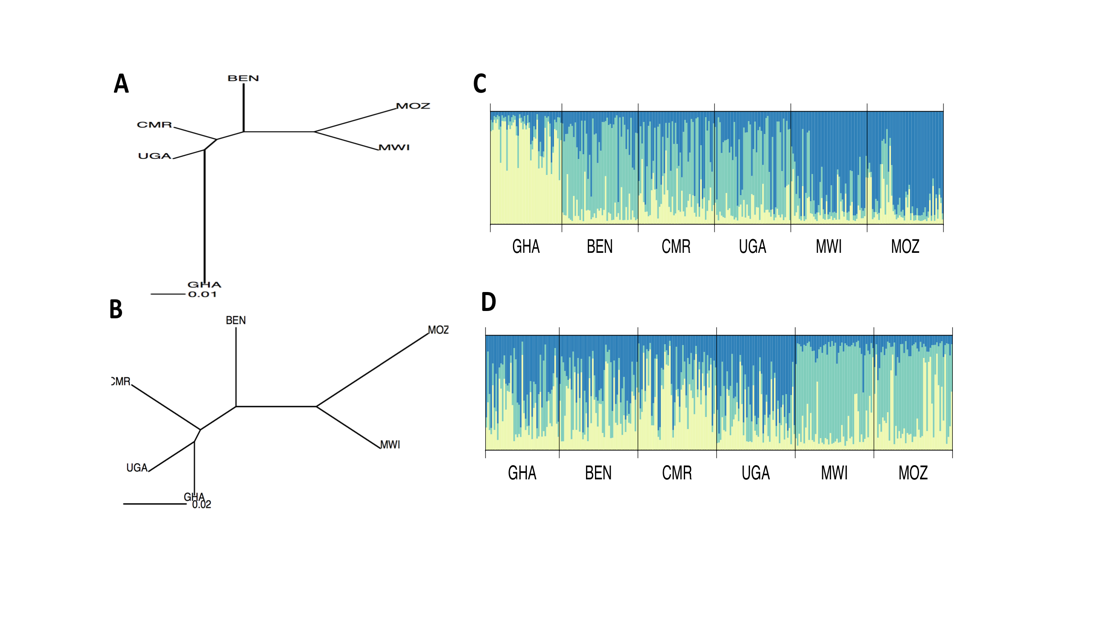

Supplement: S1 Fig — (A) Neighbor-joining tree based on the Fst of 8 microsatellites on 2R. (B) Neighbor-joining tree based on the Fst of 8 microsatellites non 2R chromosome markers. (C) Bayesian population structure of Africa based on 8 microsatellites spanning rp1 QTL on 2R chromosome: BEN-Benin, CMR = Cameroon, GHA = Ghana, MWI = Malawi, MOZ = Mozambique, UGA = Uganda. (D) Bayesian population structure of other 8 microsatellites from other chromosomes apart 2R, in order to assess how the rp1 markers are skewing the population structure. (TIFF) [file pgen.1006539.s001.tiff]

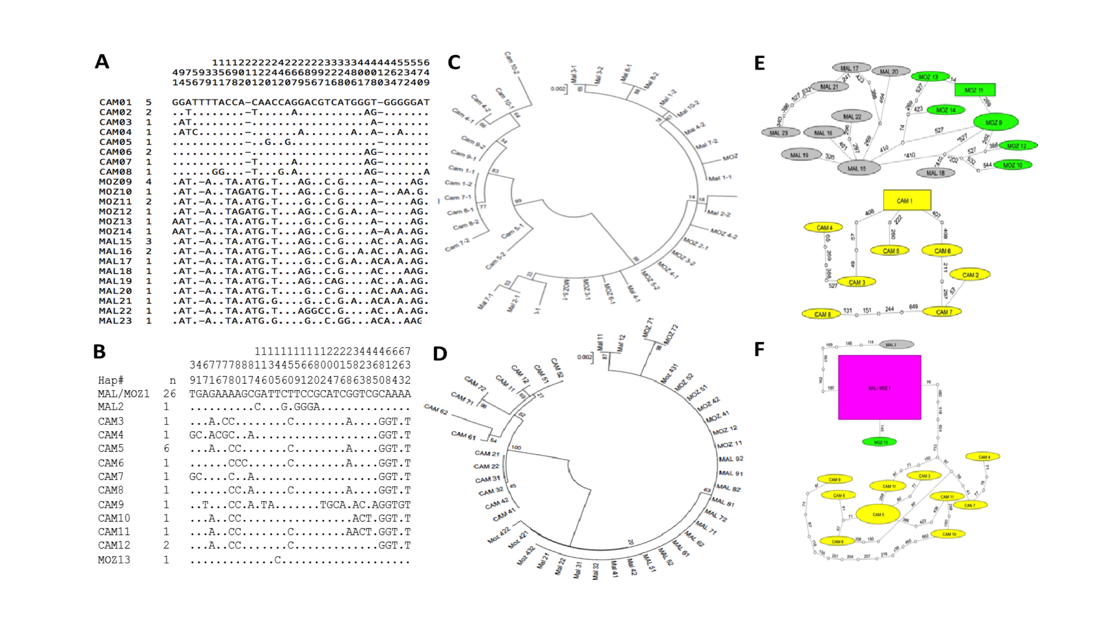

Supplement: S2 Fig — Analysis of the rp1 QTL BAC loci 25 (A) and BAC 70 (B) across the more resistant population (MAL—Malawi and MOZ—Mozambique) and the more susceptible samples (CAM—Cameroon). The polymorphic positions are indicated with and the second numbers (n) indicates the haplotype frequency. (C) Maximum likelihood tree of fragment at -34kb of CYP6P9a (BAC0) and (D) is for the fragment at -9kb of CYP6P9a (BAC 25). (E) is the haplotype network of BAC0 (-34kb) and (F) is for BAC25 (-9kb) where pink represents haplotype dominant in both Malawi and Mozambique. The size of the polygon reflects the frequency of the haplotype. Segregating mutation is represented by each node and polymorphic positions are given above the branches. (TIFF) [file pgen.1006539.s002.tiff]

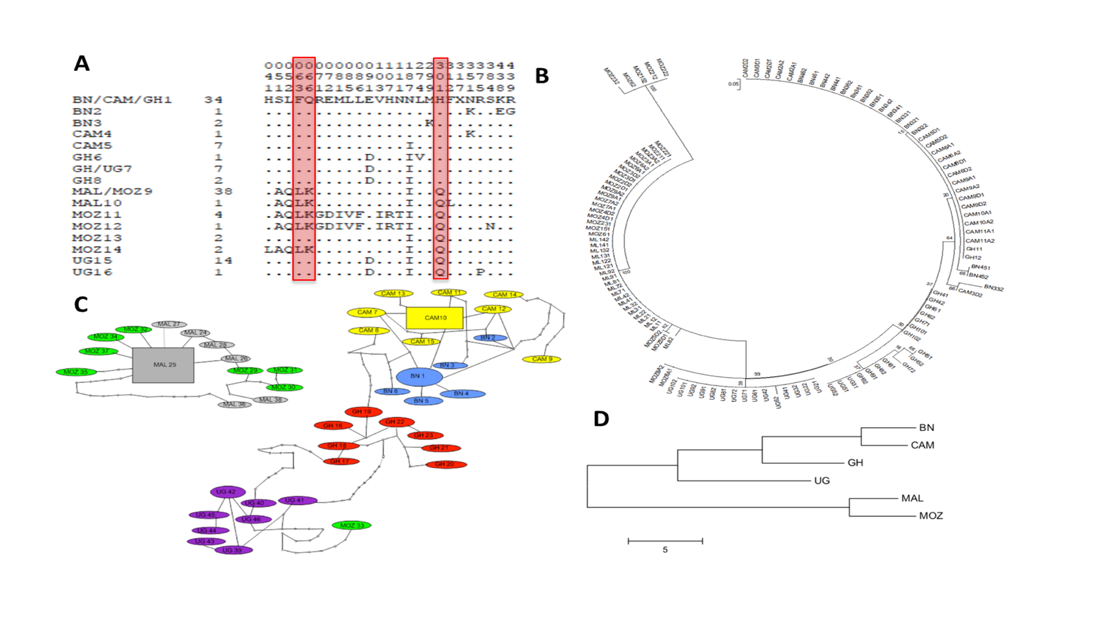

Supplement: S3 Fig — (A) Haplotype distribution of non-synonymous equivalent of amino acid protein variants. Highlighted in red are the amino acid changes linked to pyrethroid resistance [20]. (B) Maximum likelihood tree of the CYP6P9a gene (non-synonymous) changes) for six samples (BN—Benin, CAM—Cameroon, GH—Ghana, MAL—Malawi, MOZ—Mozambique and UG—Uganda). (C) Haplotype network of CYP6P9a for individual countries for coding region. The size of the polygon reflects the frequency of the haplotype and colour represents the countries (BN (Benin)–Blue, CAM (Cameroon)–Yellow, GH (Ghana)–Red, MAL (Malawi)—Grey, MOZ (Mozambique)—Green and UG (Uganda)—Purple). Segregating mutation is represented by each node and rectangular boxes represent major haplotype. (D) Neighbour joining tree based on genetic distances from KST estimates of pairwise population comparison. (TIFF) [file pgen.1006539.s003.tiff]

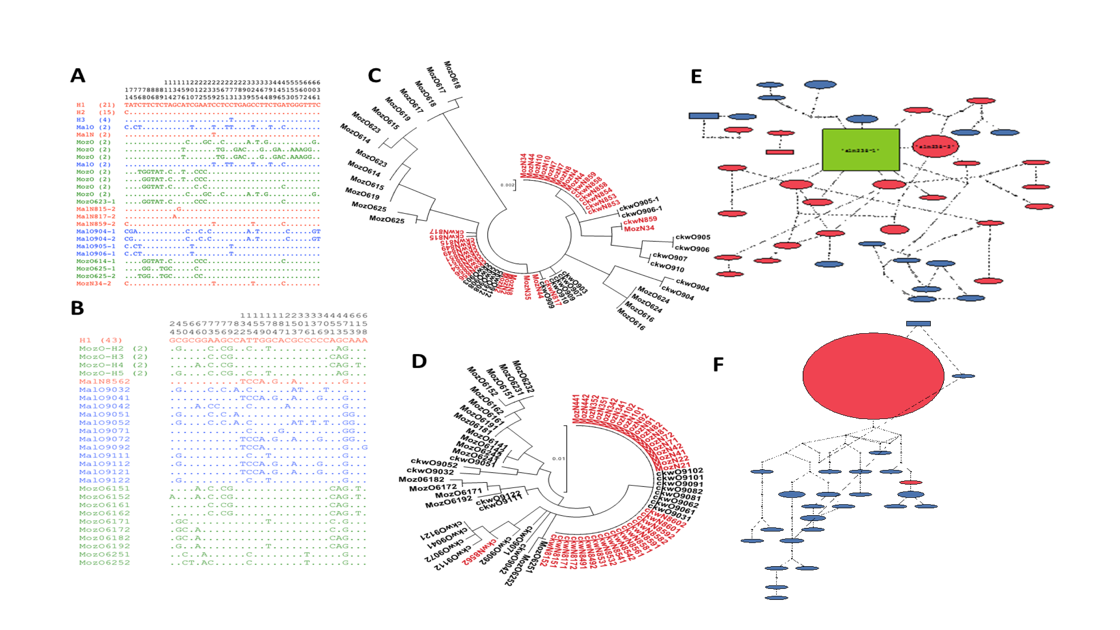

Supplement: S4 Fig — Analysis of two BAC sequences from southern Africa BAC0 on the 5’UTR and BAC25 near the gene CYP6P9a. (A-B), Haplotypes from post-intervention samples (red) and pre-intervention from Malawi (blue), and Mozambique (green) including the frequency in brackets and the SNP location on the top x-axis. (C-D), ML-tree of pre-intervention (black) and post-intervention (red) show more divergence at BAC0 while BAC25 shows distinct grouping between pre and post. (E-F), Haplotype network where the size correlated to frequency. The red denotes samples collected post-intervention, blue were collected pre-intervention and green contains sequences from both time points. (TIFF) [file pgen.1006539.s004.tiff]

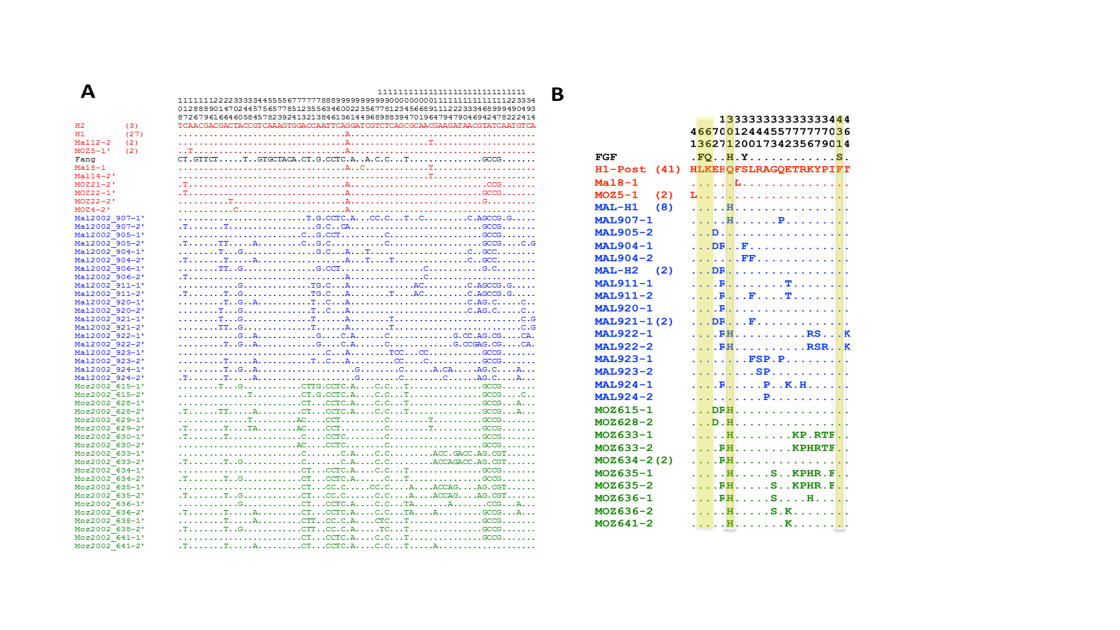

Supplement: S5 Fig — (A) All SNPs in pre-intervention samples (blue and green) and post-intervention (red) compared to a susceptible lab strain, Fang (black) including frequency in brackets and the position on the top x-axis. (B) Haplotypes based on amino acid changes pre (blue and green) versus post-intervention (red) shows a major loss of diversity. SNPs highlighted in yellow denote changes implicated in increased catalytic function [20]. (TIFF) [file pgen.1006539.s005.tiff]

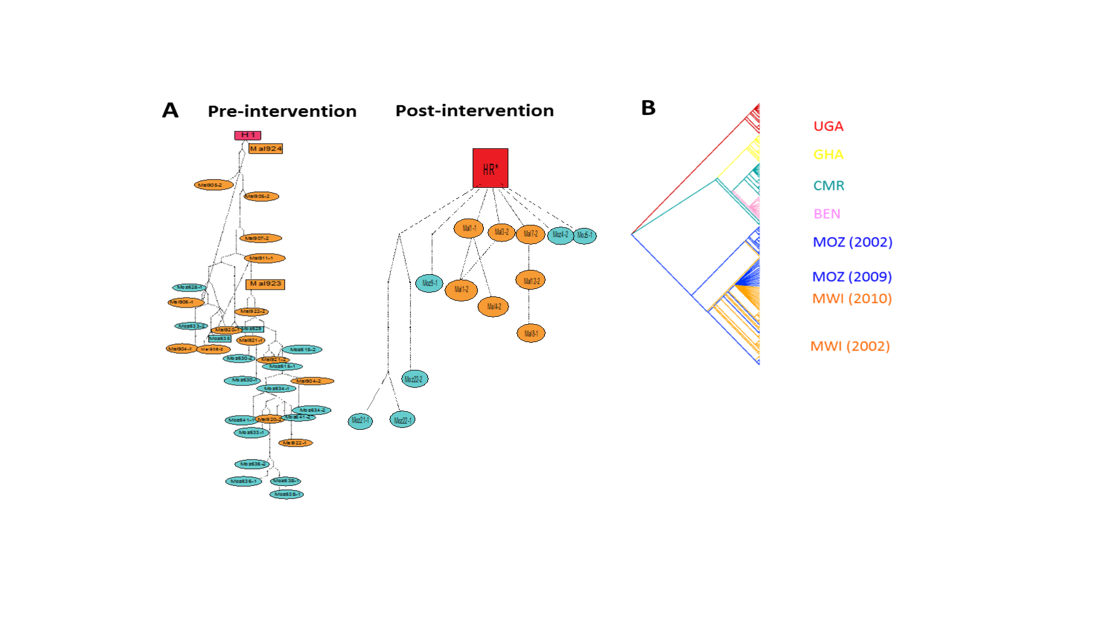

Supplement: S6 Fig — (A) The TCS haplotype network of pre- versus post-intervention samples based on the coding region including haplotypes with more than one sequence (red) and singular haplotypes from Malawi (orange) and Mozambique (blue). (B) Africa-wide Neighbor-Joining tree of CYP6P9a shows geographical clustering where southern Africa (blue and orange) is divergent. Within the southern Africa cluster there is a lack of diversity in Mozambique (MOZ 2009) and Malawi (MWI 2010). (TIFF) [file pgen.1006539.s006.tiff]

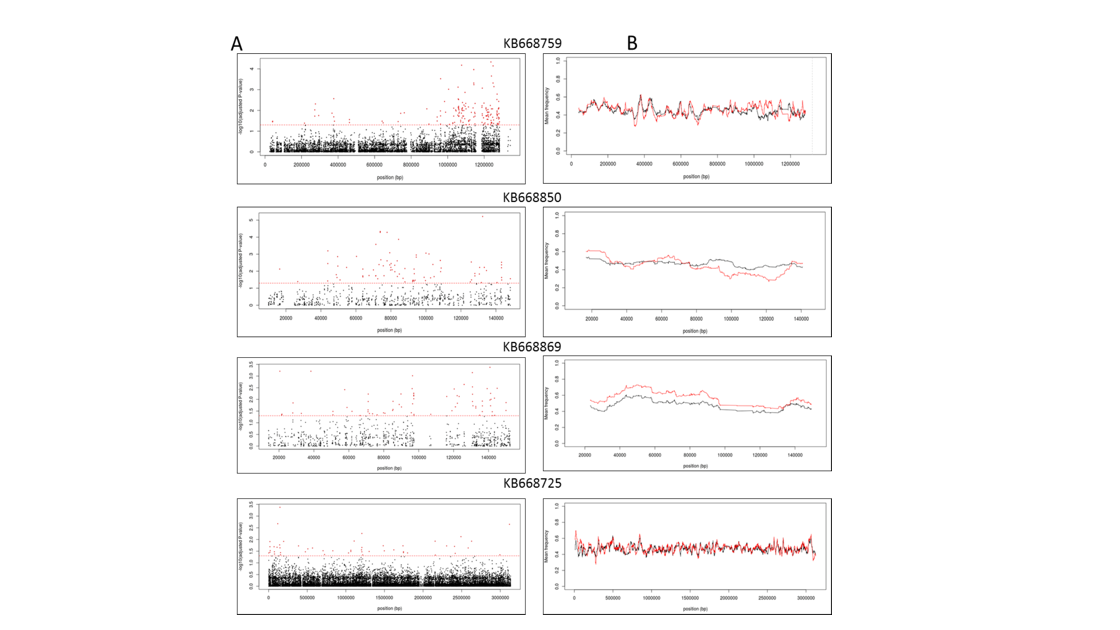

Supplement: S7 Fig — These show no striking valley of reduced variability in contrast to KB669169 spanning rp1. (A) Plot of P-values of the difference in allele frequency for sites on respective scaffold. (B) Mean frequency of non-reference alleles (for 101 sites) on respective scaffold. The black line is for MWI-2002 and the red line for MWI-2014. (TIFF) [file pgen.1006539.s007.tiff]

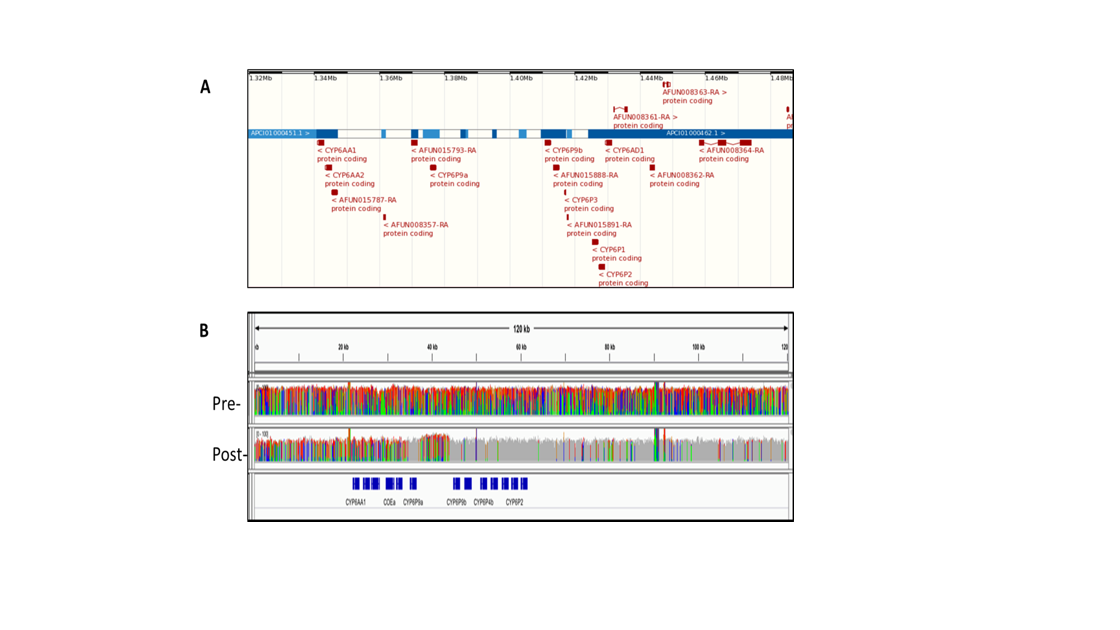

Supplement: S8 Fig — (A) Gene annotation of the rp1 region view from the Vectobase screenshot. The region from approximately 1.32 to 1.48 Mb corresponds to the sequence BAC containing rp1. Blue bars represent scaffolded contigs and spaces are unsequenced assembly gaps, which are common in this region. Red boxes represent annotated genes. (B) Contrasting polymorphism patterns between pre- and post-intervention samples: Data aligned to BAC (IGV screenshot). Full-length (120kb) BAC sequence. The top track shows the position on the BAC, the second and third show alignment depth (on a log scale for display purposes) for 2002 and 2014, respectively (coverage depth is capped at >100x). Grey columns represent bases identical to the reference sequence while coloured columns indicate variant sites with a minor allele frequency >10%. The fourth track shows genes of the P450 cluster. (TIFF) [file pgen.1006539.s008.tiff]
