# Supplementary material for: Genomic Footprints of Selective Sweeps from Metabolic Resistance to Pyrethroids in African Malaria Vectors Are Driven by Scale up of Insecticide-Based Vector Control
Source: PLoS Genet. 2017 Feb 2;13(2):e1006539. doi: 10.1371/journal.pgen.1006539 (PMC5289422; doi:10.1371/journal.pgen.1006539)
Supplement: S1 Table — (PDF) [file pgen.1006539.s009.pdf]

S1 Table: Polymorphism at 16 microsatellite loci in *An. funestus* collected from six African countries. Alleles: number of alleles in the sample, Genotyped: number of individuals genotyped, Hobs: observed proportion of heterozygotes, Hexp: Expected heterozygosity (Nei 1978), F_{IS}: inbreeding coefficient, calculated according to Weir and Cockerham (1984). Significant deviation from Hardy-Weinberg equilibrium is shown by F_{is}, with significant values in bold (* p<0.05, ** p<0.01), after Bonferroni correction for multiple testing (Holm 1979).

Marker		Ghana (n=45)	Benin (n=48)	Cameroon (n=48)	Uganda (n=48)	Malawi (n=48)	Mozambique (n=48)	Malawi Post- (2010)	Mozambique Post- (2010)	Malawi Pre- (2002)	Mozambique Pre- (2002)
AFND12	Alleles	-	-	-	-	-	-	1	1	3	3
	Genotyped	-	-	-	-	-	-	36	38	41	45
	Hobs	-	-	-	-	-	-	0.00	0.00	0.20	0.27
	Hexp	-	-	-	-	-	-	0.00	0.00	0.38	0.36
	Fis	-	-	-	-	-	-	-	-	0.48	0.27
FunQ	Alleles	5	5	6	5	7	5	1	1	3	2
	Genotyped	37	45	44	41	48	47	36	38	41	45
	Hobs	0.43	0.33	0.36	0.41	0.23	0.13	0.00	0.00	0.10	0.02
	Hexp	0.55	0.64	0.74	0.74	0.48	0.49	0.00	0.00	0.10	0.02
	Fis	0.21	0.48	0.51	0.44	0.52	0.74	-	-	-0.03	-0.00
AFUB3	Alleles	11	-	11	12	8	4	-	-	-	-
	Genotyped	41	-	46	46	43	48	-	-	-	-
	Hobs	0.76	-	0.72	0.59	0.53	0.40	-	-	-	-
	Hexp	0.79	-	0.74	0.80	0.64	0.60	-	-	-	-
	Fis	0.05	-	0.03	0.26	0.17	0.34	-	-	-	-
AFND40	Alleles	7	6	7	5	4	5	5	4	4	4
	Genotyped	37	44	46	41	39	46	36	38	41	45
	Hobs	0.70	0.77	0.61	0.71	0.44	0.41	0.53	0.21	0.46	0.44
	Hexp	0.72	0.76	0.69	0.73	0.71	0.75	0.68	0.69	0.64	0.67
	Fis	0.03	-0.02	0.12	0.04	0.38	0.45	0.22	0.69**	0.28	0.34**
AFUB6	Alleles	3	3	3	2	4	5	4	2	2	3
	Genotyped	45	46	44	48	41	41	6	38	41	45
	Hobs	0.18	0.09	0.27	0.73	0.20	0.46	0.44	0.37	0.44	0.44
	Hexp	0.21	0.08	0.32	0.47	0.25	0.59	0.37	0.42	0.47	0.41
	F_{is}	0.14	-0.03	0.14	-0.57	0.21	0.21	-0.20	0.12	0.07	-0.08
FunR	Alleles	5	6	4	6	6	4	2	2	3	3
	Genotyped	43	47	41	46	48	47	36	38	41	45
	Hobs	0.40	0.45	0.51	0.67	0.19	0.19	0.14	0.00	0.22	0.18
	Hexp	0.52	0.45	0.54	0.57	0.38	0.35	0.13	0.10	0.60	0.59
	F_{is}	0.24	0.02	0.04	-0.18	0.51	0.45	-0.06	1.00*	0.64**	0.70**
AFND6	Alleles	5	12	7	10	7	10	8	12	11	5
	Genotyped	39	43	40	40	42	41	36	38	41	45
	Hobs	0.38	0.67	0.48	0.63	0.38	0.29	0.39	0.42	0.54	0.13
	Hexp	0.70	0.85	0.77	0.82	0.66	0.82	0.70	0.81	0.81	0.69
	Fis	0.45	0.20	0.38	0.24	0.42	0.64	0.45	0.48**	0.34**	0.81**

AFND30	Alleles	8	9	8	11	9	4	8	7	7	4
	Genotyped	43	43	43	44	44	47	36	38	41	45
	Hobs	0.63	0.44	0.53	0.36	0.70	0.51	0.25	0.53	0.46	0.07
	Hexp	0.75	0.87	0.82	0.82	0.78	0.63	0.82	0.79	0.80	0.71
	Fis	0.16	0.49	0.35	0.55	0.10	0.20	0.69**	0.33*	0.42**	0.91**
AFND5	Alleles	-	-	-	-	-	-	2	5	4	3
	Genotyped	-	-	-	-	-	-	36	38	41	45
	Hobs	-	-	-	-	-	-	0.36	0.26	0.49	0.56
	Hexp	-	-	-	-	-	-	0.44	0.47	0.66	0.64
	Fis	-	-	-	-	-	-	0.18	0.44**	0.26	0.13
AFND32	Alleles	9	9	9	10	10	6	6	6	6	5
	Genotyped	41	43	41	42	43	44	36	38	41	45
	Hobs	0.49	0.65	0.80	0.71	0.65	0.68	0.69	0.58	0.73	0.49
	Hexp	0.83	0.84	0.85	0.87	0.69	0.66	0.69	0.66	0.69	0.71
	Fis	0.42	0.22	0.06	0.18	0.06	-0.03	-0.01	0.12	-0.07	0.31
FunO	Alleles	8	7	7	8	6	6	6	2	4	3
	Genotyped	40	45	46	48	47	40	36	38	41	45
	Hobs	0.73	0.80	0.85	0.88	0.51	0.55	0.64	0.55	0.54	0.31
	Hexp	0.77	0.78	0.74	0.69	0.59	0.63	0.60	0.49	0.55	0.42
	F_{is}	0.06	-0.03	-0.15	-0.26	0.14	0.13	-0.07	-0.13	0.03	0.26
AFUB11	Alleles	7	6	7	6	6	3	5	2	4	3
	Genotyped	45	47	38	46	47	48	36	38	41	45
	Hobs	0.36	0.45	0.74	0.74	0.72	0.52	0.53	0.13	0.46	0.56
	Hexp	0.50	0.46	0.75	0.58	0.59	0.47	0.64	0.49	0.54	0.51
	F_{is}	0.29	0.03	0.02	-0.28	-0.23	-0.11	0.18	0.73**	0.14	-0.09
FunL	Alleles	12	12	12	7	7	-	6	8	8	7
	Genotyped	39	46	48	43	47	-	36	38	41	45
	Hobs	0.69	0.72	0.73	0.58	0.66	-	0.58	0.50	0.61	0.47
	Hexp	0.86	0.90	0.84	0.72	0.68	-	0.72	0.72	0.81	0.65
	F_{is}	0.19	0.20	0.14	0.19	0.03	-	0.19	0.31**	0.25*	0.29*
AFUB10	Alleles	7	9	9	13	5	6	5	6	5	4
	Genotyped	39	45	46	46	42	42	36	38	41	45
	Hobs	0.54	0.73	0.52	0.65	0.64	0.45	0.58	0.42	0.73	0.47
	Hexp	0.83	0.80	0.80	0.88	0.73	0.66	0.68	0.59	0.56	0.52
	F_{is}	0.35	0.09	0.35	0.26	0.12	0.31	0.14	0.28*	-0.30	0.10
AFND7	Alleles	6	8	8	6	8	7	7	6	6	6
	Genotyped	30	47	48	44	46	43	36	38	41	45
	Hobs	0.57	0.70	0.77	0.66	0.61	0.56	0.67	0.63	0.78	0.78
	Hexp	0.68	0.69	0.81	0.62	0.72	0.65	0.75	0.66	0.73	0.68
	Fis	0.17	-0.02	0.05	-0.06	0.15	0.14	0.11	0.04	-0.06	-0.15

AFND19	Alleles<br>Genotyped<br>Hobs<br>Hexp<br>F_{is}	6<br>43<br>0.65<br>0.71<br>0.09	8<br>46<br>0.70<br>0.76<br>0.09	7<br>46<br>0.54<br>0.64<br>0.15	7<br>48<br>0.75<br>0.68<br>-0.11	4<br>46<br>0.72<br>0.73<br>0.02	8<br>47<br>0.57<br>0.51<br>-0.13	8<br>36<br>0.36<br>0.69<br>0.48	6<br>38<br>0.39<br>0.52<br>0.25	11<br>41<br>0.54<br>0.81<br>0.34**	5<br>45<br>0.24<br>0.32<br>0.23
FunF	Alleles<br>Genotyped<br>Hobs<br>Hexp<br>F_{is}	6<br>41<br>0.80<br>0.77<br>-0.05	6<br>46<br>0.54<br>0.51<br>-0.06	7<br>48<br>0.77<br>0.76<br>-0.01	8<br>47<br>0.40<br>0.79<br>0.49	6<br>43<br>0.49<br>0.50<br>0.02	7<br>44<br>0.71<br>0.70<br>-0.01	5<br>36<br>0.64<br>0.70<br>0.09	5<br>38<br>0.74<br>0.70<br>-0.05	6<br>41<br>0.66<br>0.66<br>0.00	5<br>45<br>0.64<br>0.71<br>0.10
AFUB12	Alleles<br>Genotyped<br>Hobs<br>Hexp<br>F_{is}	4<br>44<br>0.48<br>0.50<br>0.04	3<br>48<br>0.54<br>0.45<br>-0.21	4<br>47<br>0.55<br>0.60<br>0.07	5<br>45<br>0.38<br>0.47<br>0.20	3<br>45<br>0.67<br>0.57<br>-0.16	4<br>45<br>0.42<br>0.57<br>0.27	3<br>36<br>0.47<br>0.64<br>0.26	4<br>38<br>0.47<br>0.69<br>0.31*	3<br>41<br>0.32<br>0.37<br>0.13	4<br>45<br>0.36<br>0.60<br>0.40*
