# Supplementary material for: Genomic Footprints of Selective Sweeps from Metabolic Resistance to Pyrethroids in African Malaria Vectors Are Driven by Scale up of Insecticide-Based Vector Control
Source: PLoS Genet. 2017 Feb 2;13(2):e1006539. doi: 10.1371/journal.pgen.1006539 (PMC5289422; doi:10.1371/journal.pgen.1006539)
Supplement: S2 Table — (PDF) [file pgen.1006539.s010.pdf]

S2 Table: Genetic Differentiation between six African populations of *An. funestus:* Pairwise F_{st} scores per locus of populations from Ghana (GHA), Benin (BEN), Cameroon (CMR), Uganda (UGA), Malawi (MWI) and Mozambique (MOZ). All pairwise comparisons showing population structure divergence ($F_{st} > 0.05$) are bold and underlined.

Marker(s)	Chr	GHA - BEN	GHA - CMR	GHA - UGA	GHA - MWI	GHA - MOZ	BEN - CMR	BEN - UGA	BEN - MWI	BEN - MOZ	CMR - UGA	CMR - MWI	CMR - MOZ	UGA - MWI	UGA - MOZ	MWI - MOZ
FunQ	X	**0.052**	**0.150**	**0.080**	0.009	0.041	0.041	0.010	**0.055**	0.025	0.000	**0.177**	**0.143**	**0.116**	**0.096**	0.000
AFUB3	2R	n/a	0.001	-0.007	0.027	0.037	n/a	n/a	n/a	n/a	0.002	0.024	0.040	0.028	0.049	0.001
AFND40	2R	0.002	0.002	0.013	-0.009	-0.005	0.007	0.005	-0.002	-0.004	0.045	0.011	0.015	-0.003	0.004	-0.008
AFUB6	2R	**0.855**	**0.736**	**0.656**	**0.769**	**0.553**	**0.062**	**0.288**	0.036	**0.189**	**0.107**	0.002	**0.052**	**0.148**	**0.066**	**0.088**
FunR	2R	**0.274**	**0.183**	**0.220**	**0.357**	**0.348**	0.010	0.042	0.005	-0.003	0.039	**0.052**	0.035	**0.063**	**0.070**	-0.005
AFND6	2R	0.046	0.044	0.005	**0.062**	**0.092**	0.046	0.016	**0.137**	**0.088**	0.024	**0.085**	0.043	**0.057**	0.036	**0.058**
AFND30	2R	**0.094**	0.019	0.014	0.018	0.073	0.032	0.027	**0.068**	**0.126**	0.007	0.007	**0.054**	0.028	**0.065**	0.028
AFND32	2R	0.023	0.019	0.042	**0.163**	**0.146**	0.013	0.021	**0.090**	**0.056**	0.018	**0.142**	**0.132**	**0.090**	**0.106**	**0.087**
FunO	2R	0.027	-0.001	0.007	0.025	0.045	**0.054**	0.035	**0.082**	**0.078**	0.018	0.026	**0.073**	0.006	0.027	0.045
AFUB11	2L	0.006	**0.089**	**0.068**	0.046	0.042	**0.109**	0.043	0.028	0.010	0.043	0.047	**0.111**	0.002	0.027	0.008
FunL	2L	0.028	-0.007	0.026	**0.084**	n/a	0.040	**0.075**	**0.099**	n/a	0.023	**0.070**	n/a	0.035	n/a	n/a
AFUB10	2L	0.033	0.006	0.002	**0.061**	**0.074**	0.022	0.046	**0.140**	**0.152**	0.012	**0.123**	**0.152**	**0.075**	**0.099**	0.027
AFND7	3R	**0.133**	**0.058**	0.008	**0.163**	**0.227**	0.033	**0.117**	0.000	0.012	0.071	0.037	**0.079**	**0.160**	**0.210**	0.002
AFND19	3R	0.006	**0.056**	0.035	**0.105**	**0.111**	0.016	0.022	**0.062**	**0.080**	0.037	**0.104**	**0.052**	**0.088**	**0.087**	**0.102**
FunF	3L	**0.145**	0.012	0.033	**0.146**	0.011	**0.213**	**0.091**	0.048	**0.201**	**0.083**	**0.216**	0.010	**0.093**	**0.084**	**0.201**
AFUB12	3L	-0.008	0.030	0.007	0.004	**0.134**	0.048	0.007	0.016	**0.162**	**0.083**	0.003	0.025	0.033	**0.211**	**0.078**
8 markers (2R)	2R	**0.219**	**0.151**	**0.144**	**0.204**	**0.169**	0.030	0.046	**0.069**	**0.074**	0.028	0.049	**0.058**	0.048	**0.053**	0.037
3 markers (2L)	2L	0.025	0.025	0.029	**0.065**	**0.060**	**0.051**	**0.056**	**0.097**	**0.099**	0.024	**0.083**	**0.134**	0.042	**0.070**	0.019
2 markers (3R)	3R	**0.065**	**0.057**	0.024	**0.131**	**0.169**	0.025	**0.069**	0.033	0.047	**0.055**	**0.069**	**0.067**	**0.124**	**0.152**	**0.053**
2 markers (3L)	3L	**0.084**	0.020	0.023	**0.084**	**0.069**	**0.146**	**0.058**	0.032	**0.183**	**0.083**	**0.126**	0.017	**0.067**	**0.141**	**0.144**
All 16 markers	all	**0.137**	**0.100**	**0.092**	**0.146**	**0.137**	**0.050**	**0.051**	**0.065**	**0.086**	0.036	**0.077**	**0.070**	**0.064**	**0.084**	**0.051**
14 markers (no AFUB6 and FunR)	all	0.047	0.032	0.023	**0.070**	**0.081**	**0.052**	0.041	**0.069**	**0.086**	0.033	**0.080**	**0.073**	**0.060**	**0.086**	**0.051**
9 markers (no AFUB6 and FunR)	all	0.049	0.041	0.029	**0.076**	**0.088**	**0.064**	0.052	**0.065**	**0.096**	0.041	**0.093**	**0.083**	**0.071**	**0.108**	**0.063**
