# Supplementary material for: Genomic Footprints of Selective Sweeps from Metabolic Resistance to Pyrethroids in African Malaria Vectors Are Driven by Scale up of Insecticide-Based Vector Control
Source: PLoS Genet. 2017 Feb 2;13(2):e1006539. doi: 10.1371/journal.pgen.1006539 (PMC5289422; doi:10.1371/journal.pgen.1006539)
Supplement: S3 Table — (PDF) [file pgen.1006539.s011.pdf]

S3 Table: Bayesian assignment of six African populations of *An. funestus*. Proportion of assignment of each predefined population in each of the three clusters including all microsatellite markers on the 2R chromosome, markers not on the 2R chromosome and all combined markers inferred using the program STRUCTURE.

Population	Size	Cluster 1: 'Western'	Cluster 2: 'Central'	Cluster 3: 'Southern'
		8 2R-loci		
GHA	45	**0.7637**	0.1142	0.1221
BEN	48	0.1176	**0.6511**	0.2313
CMR	48	0.2102	**0.4655**	0.3243
UGA	48	0.1218	**0.5581**	0.3201
MWI	48	0.1269	0.1745	**0.6986**
MOZ	48	0.155	0.1101	**0.7349**
		8 non-2R loci		
GHA	45	**0.4447**	0.3158	0.2395
BEN	48	**0.4062**	0.3453	0.2484
CMR	48	0.3317	**0.4981**	0.1702
UGA	48	**0.5459**	0.2635	0.1906
MWI	48	0.1196	0.1775	**0.7029**
MOZ	48	0.1251	0.3244	**0.5506**
		9 loci		
GHA	45	**0.3921**	0.3587	0.2492
BEN	48	**0.5279**	0.2425	0.2296
CMR	48	0.2939	**0.5309**	0.1752
UGA	48	**0.5004**	0.2783	0.2213
MWI	48	0.1280	0.1788	**0.6933**
MOZ	48	0.1373	0.3183	**0.5444**
