# Supplementary material for: Genomic Footprints of Selective Sweeps from Metabolic Resistance to Pyrethroids in African Malaria Vectors Are Driven by Scale up of Insecticide-Based Vector Control
Source: PLoS Genet. 2017 Feb 2;13(2):e1006539. doi: 10.1371/journal.pgen.1006539 (PMC5289422; doi:10.1371/journal.pgen.1006539)
Supplement: S4 Table — (PDF) [file pgen.1006539.s012.pdf]

S4 Table: Genetic diversity parameters for 5 DNA sequences spanning the 120kb *rp1* QTL genomic region. N = number of sequences; S = number of polymorphic sites, h = number of haplotypes, π = nucleotide diversity, TjD = Tajima's D, FLD* = Fu and Li's D*. In the table, * = $p<0.05$ and ** = $p<0.01$. SLM = Salima, NKK = Nkhotakota, CKW = Chikwawa.

	2n	S	h	π	TjD	FLD*
BAC 0						
Cameroon	14	16	8	0.0063	-0.89	-1.13
SLM, Malawi	18	31	10	0.0110	-0.83	1.64
NKK, Malawi	20	29	11	0.0110	-0.66	1.65
CKW, Malawi 2002	18	19	8	0.0093	0.09	1.32
CKW, Malawi 2010	18	6	6	0.0018	-1.11	-1.47
Mozambique 2002	18	32	9	0.0210	1.50	1.66
Mozambique 2010	18	4	3	0.0013	-0.79	-1.61
BAC 25						
Cameroon	16	22	10	0.0065	-1.00	-1.10
SLM, Malawi	20	8	2	0.0010	-2.17**	-3.18**
NKK, Malawi	18	16	6	0.0027	-2.13*	-2.81**
CKW, Malawi 2002	20	22	11	0.0080	-0.02	0.13
CKW, Malawi 2010	18	7	2	0.0010	-2.10*	-2.94**
Mozambique 2002	18	15	11	0.0074	1.13	0.86
Mozambique 2010	18	0	1	n/a	-2.97**	-3.45**
BAC 63/70*						
Cameroon *	14	10	4	0.0038	-0.90	-0.26
SLM, Malawi	20	2	2	0.0005	-0.77	0.87
NKK, Malawi	18	2	2	0.0006	-0.69	0.89
CKW, Malawi 2002	18	61	10	0.0347	1.42	1.63
CKW, Malawi 2010	22	27	6	0.0060	-1.63	0.16
Mozambique 2002	18	53	9	0.0224	0.12	1.71
Mozambique 2010	16	9	2	0.0051	1.20	1.39
BAC 95						
Cameroon	12	13	10	0.0040	-1.66	-1.76
SLM, Malawi	20	7	5	0.0036	0.81	0.67
NKK, Malawi	18	10	4	0.0031	-1.01	1.41
CKW, Malawi 2002	20	17	15	0.0057	-0.70	-0.91
CKW, Malawi 2010	18	8	6	0.0020	-1.43	-0.84
Mozambique 2002	20	40	15	0.0240	1.73	1.55
Mozambique 2010	18	11	6	0.0036	-0.86	1.01
BAC 120						
Cameroon	10	10	7	0.0041	-1.43	-1.25
SLM, Malawi	18	12	4	0.0076	1.69	1.46
NKK, Malawi	20	18	7	0.0056	-1.13	-0.10
CKW, Malawi 2002	18	24	8	0.0163	1.96	1.40
CKW, Malawi 2010	18	15	7	0.0035	-1.76	-1.44

Mozambique 2002	18	36	10	0.0222	1.40	1.39
Mozambique 2010	18	29	4	0.0203	2.42*	1.63
