# Supplementary material for: Genomic Footprints of Selective Sweeps from Metabolic Resistance to Pyrethroids in African Malaria Vectors Are Driven by Scale up of Insecticide-Based Vector Control
Source: PLoS Genet. 2017 Feb 2;13(2):e1006539. doi: 10.1371/journal.pgen.1006539 (PMC5289422; doi:10.1371/journal.pgen.1006539)
Supplement: S5 Table — (PDF) [file pgen.1006539.s013.pdf]

S5 Table: Genetic parameters for *CYP6P9a* across Africa and between pre- and post-intervention samples. Genetic parameters of the full-length sequence followed by the coding only then noncoding only. N = Number of sequences; S = Number of polymorphic sites; h = Number of haplotypes; Hd = Haplotype diversity; π = Nucleotide diversity (π is raised to 10^{-4}); TjD = Tajima's D; FL-D* = Fu and Li's D*. In the table, * = $p < 0.05$.

S	N	S	π	h (Hd)	Syn	NSyn	TjD	FLD*	S	π	h (Hd)	TjD	FLD*	S	π	h (Hd)	TjD	FLD*
Pre-intervention																		
MAL	32	56	8.4	20(1)	34	17	0.17	0.79	51	10.1	32(1)	0.61	1.71	17	0.78	19(0.95)	-0.24	0.85
MOZ	20	48	7.3	20(1)	25	11	0.26	1.24	35	7.1	19(0.99)	0.11	1.21	14	8.4	12(0.95)	0.25	1.13
All-Pre	52	72	10	52(1)	35	20	0.95	1.94*	54	10.5	51(0.99)	0.94	2.01*	18	10.1	29(0.97)	0.84	1.24
Post-intervention																		
MAL	20	5	0.8	8(0.84)	2	1	-2.43*	-2.39*	3	0.5	5(0.621)	-1.94*	-1.84	2	1.5	3(0.65)	-1.12	-0.99
MOZ	20	13	1.1	8(0.7)	7	1	-2.54*	-2.25*	8	0.9	6(0.51)	-1.97*	-2.05*	5	1.5	5(0.51)	-1.39	-1.76
All-Post	40	17	0.98	14(0.78)	9	2	-2.44*	-2.29*	11	0.75	10(0.57)	-1.74	-1.85	6	1.6	6(0.61)	-1.11	-1.37
Across Africa																		
BN	16	25	2.2	11(0.908)	8	4	-1.72	-1.58	12	1.2	6(0.617)	-1.89	-1.95	13	5.1	10(0.83)	-1.35	-0.99
CAM	20	34	2.9	10(0.832)	24	1	-1.57	-1.15	25	2.9	9(0.789)	-1.49	-0.79	9	2.9	5(0.568)	-1.47	-1.72
GH	16	14	2.0	9(0.917)	8	4	-0.44	-0.06	11	2.0	8(0.883)	-0.69	-0.05	3	2.1	5(0.758)	-0.47	-0.04
UG	18	16	1.5	10(0.908)	5	2	-1.34	-0.95	7	1.2	8(0.837)	-0.38	0.70	9	2.4	4(0.399)	-1.92*	-2.11*
Total	162	140	14.6	106(0.98)	67	33	0.39	0.06	100	14.7	91(0.96)	0.57	0.61	40	14.1	58(0.95)	-0.06	-1.1
