# Supplementary material for: Genomic Footprints of Selective Sweeps from Metabolic Resistance to Pyrethroids in African Malaria Vectors Are Driven by Scale up of Insecticide-Based Vector Control
Source: PLoS Genet. 2017 Feb 2;13(2):e1006539. doi: 10.1371/journal.pgen.1006539 (PMC5289422; doi:10.1371/journal.pgen.1006539)
Supplement: S6 Table — (PDF) [file pgen.1006539.s014.pdf]

**S6 Table: Genetic differentiation ($K_{ST}$) based on polymorphism in the CYP6P9a gene.**

| | **Benin** | **Cameroon** | **Ghana** | **Malawi** | **Mozambique** |
|---|---|---|---|---|---|
| **Cameroon** | 0.14494*** | | | | |
| **Ghana** | 0.53047*** | 0.47588*** | | | |
| **Malawi** | 0.34696*** | 0.64195*** | 0.49400*** | | |
| **Mozambique** | 0.56865*** | 0.58241*** | 0.57271*** | 0.04823* | |
| **Uganda** | 0.52071*** | 0.62288*** | 0.43902*** | 0.48077*** | 0.57465*** |

PERMTEST calculates Hudson's $K_{ST}$ statistic of genetic differentiation. $K_{ST}$ is equal to $12K_{S}/K_{T}$, where $K_S$ is a weighted mean of K1 and K2 (mean number of differences between sequences in subpopulations 1 and 2, respectively) and $K_T$ represents the mean number of differences between two sequences regardless of their subpopulation. The null hypothesis of no genetic differentiation will be rejected (P<0.05) when $K_S$ is small and $K_{ST}$ is close to 1. PM test; Probability obtained by the permutation test with 1000 replicates); ns, not significant; *, 0.01<P<0.05; **, 0.001<P<0.01; ***, P<0.001
