# Supplementary material for: Genomic Footprints of Selective Sweeps from Metabolic Resistance to Pyrethroids in African Malaria Vectors Are Driven by Scale up of Insecticide-Based Vector Control
Source: PLoS Genet. 2017 Feb 2;13(2):e1006539. doi: 10.1371/journal.pgen.1006539 (PMC5289422; doi:10.1371/journal.pgen.1006539)
Supplement: S7 Table — (PDF) [file pgen.1006539.s015.pdf]

S7 Table. Descriptive statistics of POOLseq sequence read data for field-caught mosquitoes from Malawi.

Sample name	Untrimmed reads	Trimmed reads	R1/R2 pairs [1]	R0 reads (%) [2]
MWI-Chikwawa-2014	91,499,782	90,976,162	45,240,390	495,382 (0.54%)
MWI-Chikwawa-2002	100,946,514	100,344,549	49,951,213	442,123 (0.44%)

[1] Forward (R1) and reverse (R2) read pairs after trimming.
[2] Reads unpaired after trimming (% of total trimmed reads).
