# Supplementary material for: Genomic Footprints of Selective Sweeps from Metabolic Resistance to Pyrethroids in African Malaria Vectors Are Driven by Scale up of Insecticide-Based Vector Control
Source: PLoS Genet. 2017 Feb 2;13(2):e1006539. doi: 10.1371/journal.pgen.1006539 (PMC5289422; doi:10.1371/journal.pgen.1006539)
Supplement: S8 Table — (PDF) [file pgen.1006539.s016.pdf]

S8 Table. Descriptive statistics of POOLseq sequence alignments and coverage depth of field-caught mosquitoes from Malawi.

Sample ID	Reads to align (R1+R2+R0)	Aligned reads (%) [1]	Aligned reads, filtered (%) [1,2]	Aligned R1 (%) [3]	Aligned R2 (%) [3]	Aligned in pair (%) [3]	Properly paired (%) [3,4]	Singleton (%) [3]	Median coverage depth	5th-95th centile	25th-75th centile
MWI-Chikwawa-2014	90,976,162	30,929,682 (34%)	28,399,605 (31%)	14,207,546 (50%)	14,107,097 (50%)	28,158,651 (99%)	24,343,506 (86%)	155,992 (0.5%)	16	8-25	13-20
MWI-Chikwawa-2002	100,344,549	82,126,137 (82%)	74,115,352 (74%)	37,042,968 (50%)	36,813,791 (50%)	73,528,738 (99%)	67,567,770 (91%)	328,021 (0.4%)	43	26-58	38-49

[1] % of reads to align.
[2] Aligned reads filtered to remove duplicates and reads with mapping quality <10.
[3] % of filtered aligned reads.
[4] Properly paired means both read and its mate are mapped to opposing strands of the reference sequence, with 3’ ends innermost and 5’ ends within the allowed distance from each other (0-500 bp
