# Supplementary material for: Genomic Footprints of Selective Sweeps from Metabolic Resistance to Pyrethroids in African Malaria Vectors Are Driven by Scale up of Insecticide-Based Vector Control
Source: PLoS Genet. 2017 Feb 2;13(2):e1006539. doi: 10.1371/journal.pgen.1006539 (PMC5289422; doi:10.1371/journal.pgen.1006539)
Supplement: S9 Table — (PDF) [file pgen.1006539.s017.pdf]

S9 Table. Numbers of SNPs before and after filtering for coverage depth for POOLseq sequence alignments of field-caught mosquitoes from Malawi, individually and both together.

Sample ID	SNP (all)	SNP (5th-95th centile)	SNP (25th-75th centile)
MWI-Chikwawa-2014	2,443,827	2,294,950	1,364,170
MWI-Chikwawa-2002	4,615,105	4,123,043	2,025,066
Both	4,705,652	3,244,415	979,808
