# Supplementary material for: Genomic Footprints of Selective Sweeps from Metabolic Resistance to Pyrethroids in African Malaria Vectors Are Driven by Scale up of Insecticide-Based Vector Control
Source: PLoS Genet. 2017 Feb 2;13(2):e1006539. doi: 10.1371/journal.pgen.1006539 (PMC5289422; doi:10.1371/journal.pgen.1006539)
Supplement: S10 Table — (PDF) [file pgen.1006539.s018.pdf]

S10 Table: Microsatellite marker information and BAC clone primer information. For Microsatellite markers, lowercase text in left primers indicates 19 basepair tails used to add dye-labelled oligos. AFND12 and AFND5 were used in the southern Africa-specific analysis while AFUB3 was used in the Africa-wide analysis. BAC clone and CYP6P9a primer information used for sequencing is listed in grey.

Marker or Primer	Chromosome (band)	Dye	Size Range	Reverse	Forward
AFND12*	X (3B)	D3	87-107	gtagtcgacaatccgtacgGTTCTCCATCGCTGTTCTACTC	TATAACGTTTCGTACACACGCC
FunQ	X (5D)	D4	238-246	atcggactcgagctaagcgGCAAACTGCTAGTAAATGTTTCC	ACATTTCCACAATTTGCGC
AFUB3*	2R (8B)	D2	164-182	cacgacgttgtagaacgacGGGAAGGATTCGACCTTAGC	GCCGCCATTTAGTAGCAGTT
AFND40	2R (9B)	D3	188-200	gtagtcgacaatccgtacgATTCATCCTGTGATGCTTTG	AGGCTCTTCTTTGCACTGT
AFUB6	2R (11A)	D3	145-151	gtagtcgacaatccgtacgCCAGCAGGTGTGGAGGAC	GTCGTACAAAAGCACCACCA
FunR	2R (12A)	D2	132-148	cacgacgttgtagaacgacGTAGTCGATGGTGCCGTGTG	ACCGTCCCTTCCATCTGTGA
AFND6	2R (12E)	D3	184-212	cacgacgttgtagaacgacGCTTCTTCTCCCCTAATCTG	TCCTGCTTTTTAGTTTGTCG
AFND30	2R (13C)	D4	81-107	gtagtcgacaatccgtacgGTTCAGCTGTTGGTGTGTTAG	TTTTCGTACGGAGAAAAATG
AFND5*	2R (15C)	D4	169-183	atcggactcgagctaagcgCCTCTCGTTGTGTTGCCTAC	GTTCATACGTTGCCCGATTT
AFND32	2R (15E)	D4	103-121	atcggactcgagctaagcgGAAGCATTTTGGGTTAGACTC	GCAGTTGTTTACCTTTCACTG
FunO	2R (18A)	D4	116-124	atcggactcgagctaagcgGCACACATTTCAGGCAGC	GCCCACATTCTGCACCTT
AFUB11	2L (20D)	D3	188-195	gtagtcgacaatccgtacgCAGTTTCTGCGTGGAGGAAT	AGCAGCTGATGAGCCATCTC
FunL	2L (24C)	D3	140-166	gtagtcgacaatccgtacgAACAGTGGAAGGCAAATTGC	GCACGGTTACCACTGCTCA
AFUB10	2L (26CD)	D4	195-210	atcggactcgagctaagcgTGTCCATGTACAACCGCAAC	TTCTCCAGCATCATCAGCAC
AFND7	3R (33A)	D2	70-84	cacgacgttgtagaacgacTGCATCATTCGACTCGGAAG	AACGGCACTACCGTTCACTG
AFND19	2L (23A)	D4	172-205	atcggactcgagctaagcgCAGAACCACTTCGATTCAAC	CCTGCACTCAGAAACACAC
FunF	3L (43A)	D3	104-118	gtagtcgacaatccgtacgGCCTTCAGTTTCGATTGGCG	AATAAGATGCGACCGTGGC
AFUB12	3L (46C)	D3	87-107	gtagtcgacaatccgtacgTGGGGAACTGGTCGTTAGAG	CTGGTGATGGGATTGAGGAT
0 BAC	2R Chromosome		716	TCACACTAGCTGCCAAATCG	TGGTAGCTGCTTGAGGAGAAA
25 BAC			823	TCATCTCCTTGAGTGCATCG	GTGACGGATCTGGACCTTGT
63 BAC			719	ACCAACCGGTAAGTTTCGTG	ATTACGAACCGTCAGCCATC
95 BAC			755	CTCCTCCGTAACGTGATCGT	CCTCTTTACTGGCCACCGTA
120 BAC			711	AGTTCGAGCACCAGCTCAAG	TCTTCCGCCATTGTGTATCA
CYP6P9a 5'			754	GCGTTTGAGTTTAATCTGATGATTATTC	ATCCCTAACTATTAAAAGGCAAT
CYP6P9a 3'			815	TCACAATTTTTCCACCTTCAAGTAATTACCCGC	GAATAATCATCAGATTAAACTCA
