# Supplementary material for: Genomic Footprints of Selective Sweeps from Metabolic Resistance to Pyrethroids in African Malaria Vectors Are Driven by Scale up of Insecticide-Based Vector Control
Source: PLoS Genet. 2017 Feb 2;13(2):e1006539. doi: 10.1371/journal.pgen.1006539 (PMC5289422; doi:10.1371/journal.pgen.1006539)
Supplement: S1 Text — (DOCX) [file pgen.1006539.s019.docx]

**Supplementary Information - Genomic footprints of selective sweeps from metabolic resistance to pyrethroids in African malaria vectors are driven by scale up of insecticide-based vector control**

**Results**

**Continent-wide genetic diversity based on a panel of microsatellites**

All individual mosquitoes used for this study were confirmed to be *An. funestus sensu stricto* by a species-typing PCR assay. Individual mosquitoes (N=45-48) from six countries from Southern (Mozambique and Malawi), East (Uganda), Central (Cameroon) and West Africa (Benin and Ghana) were used for microsatellite genotyping. Moderate to high levels of polymorphism were observed in all six populations (S1 Table and Fig. 1B). The total number of alleles genotyped ranged from 6 (AFUB6, AFUB12) to 17 (AFND6) and the mean number of alleles per population ranged from 3.3 (AFUB6) to 10 (FUNL).

Gene diversity across the genome was estimated for 11 microsatellites. The results (Fig. 1A) show gene diversity values generally range between 0.6 and 0.8. Most striking is the reduced gene diversity at two loci (AFUB6, FUNR) on chromosome arm 2R. This region contains the *rp1* pyrethroid resistance locus with the FUNR marker located at the 5'UTR region of the *CYP6P9a* pyrethroid resistance gene. Therefore, we hypothesised that the reduced gene diversity may be a result of one or more selective sweeps driven by selection pressure from pyrethroid use. Genotyping a further 5 microsatellite markers on the 2R around FUNR and AFUB6 revealed that the reduced diversity was restricted to the two markers and did not extend to all the 2R chromosome suggesting that it could be associated with pyrethroid resistance. A similar pattern is observed when Observed (Ho) and Expected (He) heterozygosity are assessed (S1 Table).

**Continent-wide population structure of *Anopheles funestus***

A significant deviation from Hardy-Weinberg equilibrium (HWE) was observed in 24 out of 94 independent tests of HWE across loci and populations (p<0.05 after Bonferroni correction). Most deviations were as a result of a deficit of heterozygotes (S1 Table). Deviations from HWE can result from inbreeding, selection or population stratification (leading to a Wahlund effect), additionally, this can be due to an artefact arising from untyped (“null”) alleles due to mutations in primer binding sites preventing amplification of some alleles. Any of these factors can result in an excess of homozygotes. No consistent pattern of linkage disequilibrium was seen among microsatellites. Levels of genetic differentiation between populations defined by geographic location were estimated using *F_ST_*. When considering 11 markers, average *F_ST_* for each microsatellite among populations ranged from 0.0204 (AFUB3) to 0.4552 (AFUB6) with highest estimates observed for *rp1* markers.

**Fine-scale analysis of selective sweep through *rp1* sequencing**

To further validate the putative selective sweep on 2R around AFUB6 and FUNR and whether it was associated with the *rp1* pyrethroid resistance QTL, a fine scale analysis of the polymorphism pattern of a 120kb genomic BAC clone spanning *rp1* was performed. This 120kb fragment includes a cluster of 15 cytochrome P450 genes among which the two duplicated P450s *CYP6P9a* and *CYP6P9b* shown to be the main drivers of pyrethroid resistance in the field. The polymorphism of five 700bp sequence regions spanning the 120kb *rp1* BAC clone was analysed from two highly resistant populations (Malawi and Mozambique) and of a moderately resistant population (Cameroon).

**Polymorphism patterns across *rp1* 120kb**: Across the five loci analysed around *CYP6P9a*, the Malawi population consistently exhibited a lower polymorphism level than Cameroon -9kb from *CYP6P9a* (BAC25) to +86kb (BAC120). The lower polymorphism in Malawi and Mozambique around *CYP6P9a* is also reflected by the genetic parameters with lower genetic diversity and haplotype diversity observed in these two countries than in Cameroon (S5 Table).

**Maximum likelihood phylogenetic trees and Haplotype network with TCS: *rp1* genomic fragments**

Construction of Maximum likelihood (ML) phylogenetic trees of the five fragments further highlighted the reduced diversity close to *CYP6P9a* in the resistant populations of Malawi and Mozambique. Where the more resistant populations shared haplotypes with the more susceptible samples, there were higher mutational steps between Malawi and Cameroon for -34 to +61 than between Malawi and Mozambique except for +86 (BAC 120). The case was the same between Mozambique and Cameroon. However, for +86, the more resistant Mozambique samples had many more mutational steps than the more susceptible Cameroon samples with several haplotypes also shared between the two samples at this position.

However, at -9kb from CYP6P9a, only one haplotype exhibits a difference of 6 mutational steps to the other two haplotypes in Malawi and Mozambique, which further supports the directional selection acting on this locus probably because of the proximity with the *CYP6P9a* resistance gene. In Cameroon, the ten haplotypes are highly diverse with more than 20 mutational steps varying between some of them, supporting an absence of selection in this sample (S3F Fig.). A similar pattern of reduced haplotype diversity is observed at +36kb and +61kb in Malawi and Mozambique with a difference of only 1 mutational step between the haplotypes of Malawi and Mozambique at the +36kb loci in contrast to those from Cameroon (>6 steps). For the +61kb loci a predominant haplotype is still present in Malawi and Mozambique with only a single haplotype from Mozambique (MOZ9) having more than one mutational step difference to other haplotype. However at the +86kb locus, reduced haplotype diversity is only observed in Malawi not in Mozambique, which is highly diverse as in the susceptible Cameroon. Indeed, if the Malawi population remains less diverse with only two haplotypes observed, a high proportion of singleton haplotypes is found in Mozambique with also more than 13 mutational steps difference between them. This diversity is even higher than in Cameroon where only 5 haplotypes are noted with only 1 haplotype having a difference of 10 mutational steps to others.

**Genetic diversity of the CYP6P9a gene across Africa**

**Polymorphism patterns of *CYP6P9a***: There were 140 polymorphic sites overall (S5 Table) and 100 polymorphic sites (33 non-synonymous) in the coding region. Analysis of polymorphism patterns revealed that *CYP6P9a* was under strong directional selection in southern African countries of Malawi and Mozambique but not for other regions.

**Maximum likelihood phylogenetic trees and Haplotype network with TCS for *CYP6P9a***

Analysis of the haplotype diversity also provided evidences of selection acting on *CYP6P9a* in southern Africa. Indeed, from a total of 59 haplotypes observed for the full *CYP6P9a* gene and 46 for the coding region (S3C Fig.), Malawi and Mozambique exhibited the lowest number of singleton haplotypes (1 and 2) in contrast to very high number in Benin and Cameroon (16 and 17) while Ghana and Uganda were intermediate (with 4 and 8). Furthermore, the frequency of the predominant haplotypes was much higher in Malawi (35%) and Mozambique (65%) for both the entire sequence and the coding sequence only, compared to other countries suggesting the selection of a specific haplotype. Interestingly, this predominant haplotype (MAL/MOZ23) in southern Africa is also the predominant resistant haplotypes previously shown to be an efficient pyrethroid metaboliser (*1*).

When considering the full gene, the resistant haplotype MAL/MOZ33 was predominant, accounting for 16.9% of the total sample, due to its high frequency in Malawi and Mozambique. This same haplotype corresponds to the resistant haplotype that have already been seen in the previously reported as important changes in the gene (*1*). When considering only the coding region, the most predominant haplotype was MAL/MOZ28, which accounted for 23.7% of the entire sample (Fig. 2D). Haplotype BN/CAM/GH/UG accounted for 5.6% of the entire dataset. This haplotype was not found Malawi or Mozambique. There were many singletons among the populations (BN-16, CAM-11, GH-4).

**Assessing the role of insecticide-based interventions in the selective sweep in southern Africa**

**Signature of selective sweep around 120kb genomic region of *rp1* in southern Africa:** To assess whether the presence of this selective sweep was associated with the implementation of insecticide-based interventions across southern Africa such as LLINs and IRS, we compared the genetic diversity of the southern Malawi and Mozambique samples collected post-interventions to the pre-intervention samples from the same locations by analysing the polymorphism spanning the 120kb *rp1* genomic region. A significant difference in polymorphism was observed with both the southern Malawi and Mozambique samples collected pre-intervention (2002) showing no reduction of diversity across the *rp1* and a high polymorphism was observed across the entire 120k region even at the -9kb from *CYP6P9a*. However, the nucleotide diversity (π) showed a greater loss of genetic diversity in post-intervention samples (Fig. 3D). This is shown for example at -9kb from CYP6P9a (BAC25) by a significant reduction of number of substitution sites from 22 and 15 respectively in Malawi and Mozambique pre-intervention in 2002 to only 7 and 1 respectively after intervention 8 years later. Similarly, a sharp reduction of the haplotype number and diversity is observed with 11 haplotypes in both locations pre-interventions but only 2 and 1 after interventions respectively in Malawi and Mozambique. A similar pattern was observed at +36kb from CYP6P9a (BAC63) and at +61kb (BAC95). There was a recovery in diversity near the ends of the *rp1* (BAC0 and BAC120) broadly showing that a selective sweep is consistently occurring around *CYP6P9a* and *CYP6P9b* throughout southern Africa, post-intervention.

### Comparative analysis of CYP6P9a genetic diversity pre and post intervention: This analysis revealed a significant loss of overall genetic diversity after intervention with 2002 samples having a total of 68 substitution sites (MAL=56; MOZ=48) while samples collected post-intervention had only 17 polymorphisms (MAL=5; MOZ=13) (S5 Table). The absence of a reduced diversity before intervention can be observed from other genetic parameters such as nucleotide diversity (π), haplotype diversity (Hd) or the number of nucleotide difference between sequences (k). Analysis of the coding region detected several amino acid changes including L63F, K66Q, H301Q and S384N. Some of these amino acid changes between the pre- and post-intervention samples such as H301Q and S384N are located in regions of the proteins with potential role in the interaction with pyrethroids and these amino acid changes have recently been shown to significantly impact the efficacy of the CYP6P9a to metabolise pyrethroids (2).

The TCS network also reveals a strong signature of selection after intervention. In pre-intervention samples 18/20 haplotypes from Malawi are unique (singleton) and 15/20 haplotypes from Mozambique are unique (S6A Fig.). Only three specimen (7.5%) share the same haplotype between the two locations (H1) (S6A Fig.). But for the post-intervention samples a significant loss of haplotype diversity is evident with the presence of a predominant haplotype at a high frequency of 67.5% (HR*) (S6A Fig.) corresponding to the resistance haplotype previously shown to be driving resistance in southern Africa (*1*). Furthermore the high haplotype diversity pre-intervention is shown by the large number of mutational steps between haplotypes in pre-intervention samples (up to 33) whereas haplotypes in post intervention samples were only separated by a maximum of 4 mutational steps for the coding region (S6A Fig.).

**Comparative whole genome sequencing-based scan of selective sweep signatures between pre- and post-intervention mosquitoes**: The numbers of sequence reads produced for each library before and after quality trimming (S7 Table) revealed a reduced amount of reads for the post- sample as confirmed by the indices describing the alignments of the reads to the FUMOZ reference genome (S8 Table). This is lower than expected and may indicate a problem with the quality of the reads or the DNA. The 40 individuals pooled were confirmed as *Anopheles funestus* using a species-specific PCR assay. BLAST searching of GenBank using a sample of the unmapped reads indicated that they may have derived from *Pseudomonas aeruginosa*. Alignment of all reads to the complete genome of *P. aeruginosa* strain M37351 (accession number CP008863.1) showed that 31,046,884 reads could be aligned to it (24,673,096 properly paired after filtering), suggesting heavy bacterial contamination to explain the low number of reads that could be aligned to the *An. funestus* genome. Analysis of the coverage depth of each library as summarised in S8 Table revealed that the depth was much lower for the 2014 library, due to the likely bacterial contamination described above with a median coverage depth of 16 and 43 respectively for 2014 and 2002.

Although a total of 4,705,652 SNPs were detected, only 979,808 variant sites from both samples were used for analysis after applying a filter to remove those at the extremes of coverage depth (S9 Table). These were tested to identify those with significantly different allele frequencies among samples. After correction for multiple testing, 3,078 variant sites (on 368 genomic scaffolds) showed significantly different allele frequencies in 2002 and 2014 as shown in Fig. 4A where the number of significant sites are plotted against total scaffold length. The two are significantly correlated (p<<0.01 for both Pearson’s and Spearman’s tests). However, a number of outlier scaffolds appear to be enriched for significant sites. The most extreme of these is scaffold KB669169, which spans the rp1 locus. Indeed, significant sites are clustered around the *rp1* region (Fig. 4B), and a striking loss of diversity between 2002 and 2014 is evident across the locus. In no other scaffold was the signature so striking (S7 Fig.) and it validates the results of microsatellite analysis.

The *rp1* locus was analysed in more detail. The region is poorly assembled in the reference genome (S8A Fig.). To analyse the *rp1* locus in more detail, data were aligned to the sequenced BAC containing *rp1* (S8B Fig.). The results confirm those from the whole genome analysis: that between 2002 and 2014, the *rp1* locus became FUMOZ-like. This appears to represent a selective sweep of the resistant FUMOZ-like haplotype through the local *An. funestus* population of Chikwawa after 2002.

**References**

1. J. M. Riveron *et al.*, Directionally selected cytochrome P450 alleles are driving the spread of pyrethroid resistance in the major malaria vector Anopheles funestus. *Proc Natl Acad Sci U S A* **110**, 252-257 (2013).

2. S. S. Ibrahim *et al.*, Allelic Variation of Cytochrome P450s Drives Resistance to Bednet Insecticides in a Major Malaria Vector. *PLoS Genet* **11**, e1005618 (2015).
